# Influencing factors and spatiotemporal heterogeneity of land-use carbon emissions in China’s urban agglomerations

**DOI:** 10.1038/s41598-025-31817-1

**Published:** 2025-12-09

**Authors:** Shaopei Chen, Xiaoshan Cai, Xinying Lian, Huixia Zhang

**Affiliations:** 1 School of Public Administration, Guangdong University of Finance and Economics, Guangzhou, China; 2 School of Culture Tourism, Guangdong University of Finance and Economics, Guangzhou, China

**Keywords:** Urban-scale assessment framework, Cross-cluster comparisons, Carbon emission intensity, Spatiotemporal variances, Sustainable development, Environmental sciences, Environmental social sciences, Environmental studies, Geography, Geography

## Abstract

In the context of global climate change and rapid urbanization, understanding the relationship between land use and carbon emissions is critical for sustainable development. This study investigates carbon emissions from land use and their intensity in China’s five major urban agglomerations, including Beijing-Tianjin-Hebei, Yangtze River Delta, Middle Reaches of Yangtze River, Pearl River Delta, and Chengdu-Chongqing, from 2000 to 2020. By integrating land-use and socio-economic data with geographic information systems, the Logarithmic Mean Divisia Index model, and the Kaya Identity, we analyze the spatiotemporal differentiation and evolution of carbon emissions and intensity, as well as the influencing factors behind these trends. Additionally, the Markov prediction method is employed to project land-use changes and carbon emission trends through 2025. The results indicate that the Pearl River Delta exhibited the fastest the most rapid increase in land-use carbon emissions during the study period, while simultaneously achieving the lowest land-use carbon emission intensity among all regions. Economic development and industrial structure served as the principal drivers of emission growth, whereas energy consumption intensity exerted a mitigating effect in all urban agglomerations. Despite the growing proportion of clean energy, the influence of energy structure on emission reduction remained limited, indicating a persistent dependence on fossil fuels. This study underscores the complex relationship between land use and carbon emissions in urban agglomerations, offering important insights for policy formulation. The findings emphasize the necessity of developing targeted strategies to reconcile economic growth with carbon reduction, thereby advancing sustainable land-use planning and supporting climate change mitigation and regional sustainability.

**Supplementary Information:**

The online version contains supplementary material available at 10.1038/s41598-025-31817-1.

## Introduction

As the world’s largest CO₂ emitter, China faces intensifying decarbonization challenges, with its carbon sink capacity declining from offsetting 30% of emissions (1980–1999) to merely 7–15% (2010–2020)^1–4^. The “Dual Carbon” goals (i.e., carbon emissions peaking by 2030 and achieving carbon neutrality by 2060)^5^ require urgent mitigation of carbon emissions from urban agglomerations, which accounted for 80% of national emissions in 2020^6–8^. This is primarily due to construction land expansion, which exhibits 55% higher emission intensity per unit area compared to fossil fuel combustion processes^9^.

Current research on land-use carbon emissions (LUCE) in China’s urban agglomerations employs a multidimensional analytical framework^10–13^, focusing on three core dimensions, including emission mechanisms, spatiotemporal patterns, and regional disparity. These studies demonstrate that intrinsic functional characteristics of urban agglomerations in China critically determine the spatiotemporal distribution of carbon emissions. For example, case studies of major urban agglomerations, such as the Yangtze River Delta and Beijing-Tianjin-Hebei regions), demonstrate that the synergistic interplay between socioeconomic activities and environmental governance policies significantly shapes LUCE trajectories, with effective intercity collaboration promoting regional green and low-carbon development^14–16^. Additionally, population agglomeration and spatial restructuring are recognized as key drivers of LUCE dynamics, while regional variations in economic foundations and urbanization stages result in diversified low-carbon transformation pathways^17–21^. To quantify these relationships, advanced analytical methods, including the Decoupling Model^22^, Kaya Identity^23^, and Logarithmic Mean Divisia Index (LMDI)^24^ model were employed, revealing nonlinear associations between LUCE and socio-economic indicators. Furthermore, predictive modeling systems incorporating FLUS^25^, InVEST^26^, and Markov chain models^27^ were employed to simulate future land-use changes and associated carbon impacts. These tools facilitate the optimization of land-use structures in urban agglomerations to improve carbon storage capacity and reduce emissions^28^. In summary, by integrating macro-level driving factors with micro-level pattern evolution, current research on LUCE in China’s urban agglomerations yields comprehensive understanding of the spatiotemporal dynamics governing land-use carbon emissions and sinks, thereby providing essential decision-making support for regional carbon neutrality strategies^29^.

However, current research on land-use carbon emissions in China’s urban agglomerations reveals three significant research gaps. First, existing studies lack standardized frameworks for cross-agglomeration comparisons and spatiotemporal modeling to track dynamic urbanization patterns. Second, predominant static analyses fail to capture phased emission mechanisms during critical processes such as industrial land conversion. Third, insufficient attention has been paid to indirect emissions from construction land and systemic impacts of land-use structural changes, particularly regarding spatial mismatches that affect emission efficiency through regional linkages. Addressing these gaps is crucial for advancing low-carbon land-use planning and achieving sustainable development goals in China’s urban agglomerations.

Therefore, this study proposes a multi-scale analysis framework (Fig. S1) of land-use carbon emissions across China’s five major urban agglomerations (Beijing-Tianjin-Hebei, Yangtze River Delta, Middle Yangtze River, Pearl River Delta, Chengdu-Chongqing) during 2000–2020. An urban-scale assessment framework is established to quantify phase-specific emission mechanisms in land conversion, and trace the impacts of structural land-use changes on regional carbon emissions. The integrated model examines carbon emission patterns across land categories, spatiotemporal variations in land-use carbon emission intensity, and key drivers including energy transitions, economic restructuring, and population dynamics.

## Methods

### Study area

**Fig. 1:**
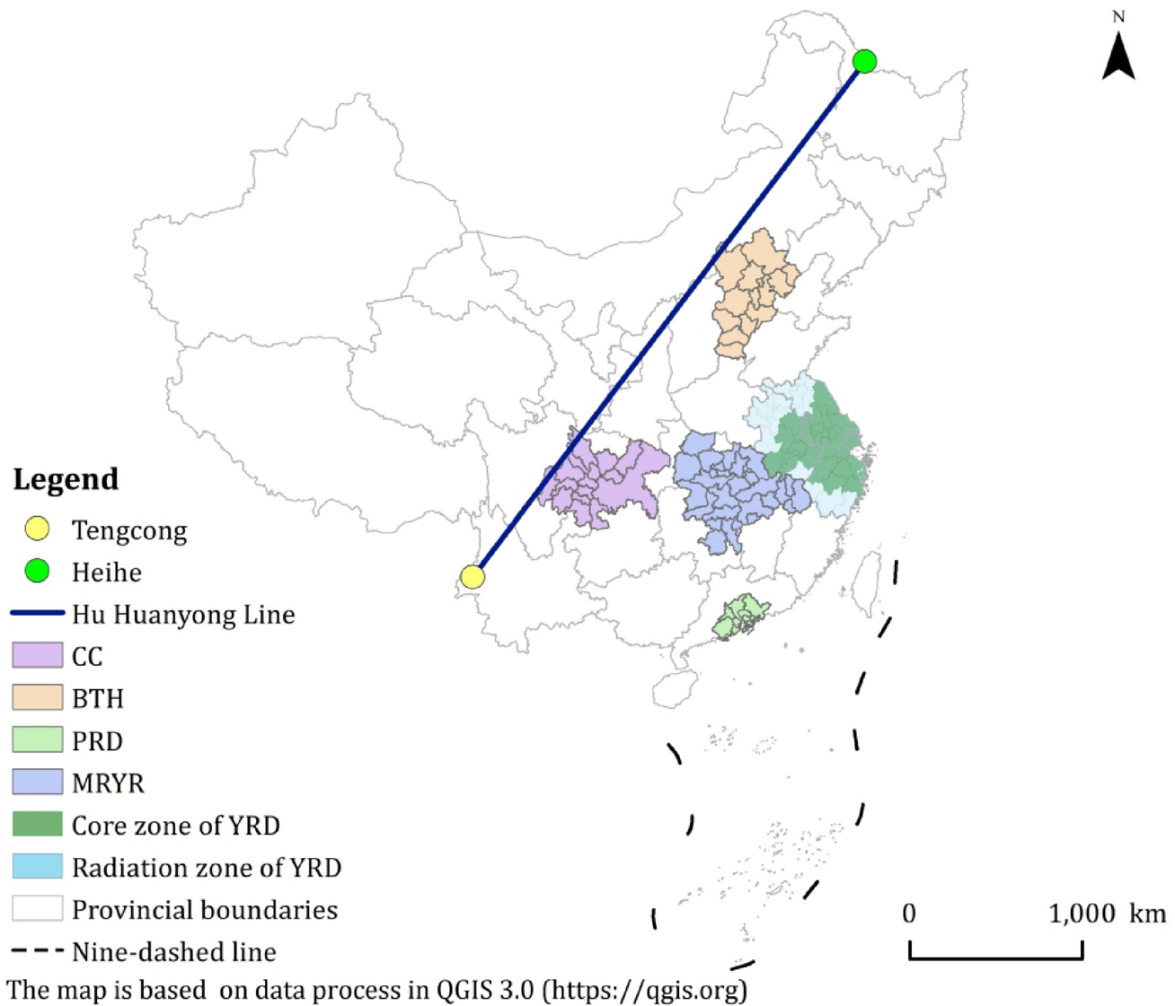
Study area.

China’s five major urban agglomerations, including Beijing-Tianjin-Hebei (BTH), Yangtze River Delta (YRD), Middle Reaches of the Yangtze River (MRYR), Pearl River Delta (PRD), and Chengdu-Chongqing (CC), serve as pivotal drivers for regional coordination and carbon neutrality initiatives, and are predominantly situated east of the Hu Huanyong Line (Fig. 1). According to the *China Statistical Yearbook of 2021*^30^, these urban agglomerations accounted for 41% of China’s population in 2020, while occupying only 10% of the national land area, and generated 59% of the national Gross Domestic Product (GDP). Notably, the YRD dominated economically, contributing 27.6 trillion yuan (20.2% of the national GDP), while also leading in CO₂ emissions, accounting for 16% of the national total^30^. (Table S1). Collectively, these urban agglomerations account for 37.8% of China’s carbon emissions, with emission patterns reflecting regional economic scale^31^. Therefore, the selection of these five urban agglomerations was based on their status as China’s most economically developed and densely populated national-level strategic regions. They collectively represent the core engines of the national economy and account for a disproportionately large share of the country’s carbon emissions, thus providing critical insights into the land-use carbon emission relationship under rapid urbanization. While other agglomerations exist east of the Hu Huanyong Line, this study focuses on these five due to their paramount national significance and comparability in scale and policy attention.

As China’s capital economic circle, the Beijing-Tianjin-Hebei (BTH) combines two municipalities directly under the Central Government (Beijing and Tianjin) and Hebei Province. In 2020, cultivated land was the dominant land-use type in BTH, accounting for 48.00% of the total land area.

The Yangtze River Delta (YRD) urban agglomeration is the most populous and economically powerful region in China. According to the *Outline of the Development Plan for Regional Integration in the Yangtze River Delta* issued in 2019, the YRD is divided into a core area and a radiation area. This study focuses on the evaluation and discussion of LUCE in the core area of YRD (Fig. 1). In 2020, the land-use composition of the YRD was as follows: 46.52% cultivated land, 26.87% forest land, 3.49% grassland, 9.05% water bodies, 13.90% construction land, and 0.16% unused land.

Centered around Wuhan, Nanchang, and Changsha, China’s largest urban agglomeration, i.e., the Middle Reaches of the Yangtze River (MRYR), showed distinct ecological emphasis: forest (49.25%) and cultivated land (36.41%) comprised over 85% of land cover.

The Pearl River Delta (PRD) urban agglomeration is the economic center of southern China, comprising nine cities in Guangdong Province. The PRD demonstrated intensive development: 53.44% forest vs. 15.05% construction land—the highest built-up ratio among peers despite contributing 12% of national GDP.

The Chengdu-Chongqing (CC) urban agglomeration, anchored by its dual cores of Chengdu and Chongqing, stands as the most rapidly expanding economic hub in western China. It exhibits striking disparities, with cultivated land accounting for 61.3% (the highest proportion in all urban agglomerations) and construction land making up just 3.93% (the lowest), underscoring its significant potential for further development.

### Data sources and preprocessing

This study integrates multi-source datasets covering the period 2000–2020 (Table 1). Land use patterns (2000–2020) were obtained from the China Land Cover Dataset (CLCD), which is sourced by the National Ecosystem Science Data Center, National Science & Technology Infrastructure of China (https://www.resdc.cn/). The CLCD dataset, developed based on Landsat 30-m imagery using a supervised Random Forest algorithm, has demonstrated robust performance with an overall accuracy of 79.31% and a Kappa coefficient of 0.76 as reported in the original publication^32^. Based on the integration of land cover classes from CLCD and the classification standards specified in GB/T 21,010 − 2017, land use is classified into six categories: cultivated land (CulL), forest land (FL), grassland (GL), water (W), construction land (ConL), and unused land (UL) (Table 2). Additionally, the administrative boundaries of provinces and prefecture-level cities and related geographical vector data, including the islands and nine-dash line data of the South China Sea, were sourced from the Resources and Environment Data Platform of the CAS.

**Table 1:** Data sources.

Data type	Data content	Data source
CLCD	Land use	The Resources and Environment Data Platform of Chinese Academy of Sciences (CAS) (https://www.resdc.cn/)
Economy development	GDP	Social and economic statistical yearbooks
	Secondary industry output value	
Society development	Population size	Social and economic statistical yearbooks
Energy	Energy consumption	Energy statistical yearbooks

**Table 2:** Land-use type and classification.

Land-use type	Primary land classification
CulL	Paddy fields, dry farmland
FL	Forested land, shrubland, sparse forest land, other forest land
GL	High, medium, and low coverage grasslands
W	Rivers and canals, lakes, reservoirs and ponds, permanent glaciers and snowfields, tidal flats, beach land
ConL	Urban land, rural residential areas, other construction land
UL	Sand land, Gobi, saline-alkali land, marshland, bare land, bare rock, texture, others

Economic and demographic data for urban agglomerations and their constituent cities were collected from statistical yearbooks covering the period 1999–2021, such as China Statistical Yearbook^33^, China Urban Statistical Yearbook^34^, and China Rural Statistical Yearbook^35^. These yearbooks contain detailed information on regional economic development, population development, industrial development, and other socio-economic variables, offering a reliable foundation for longitudinal analysis and cross-regional comparisons. Accordingly, data on GDP, population, and secondary industry output value for cities within each urban agglomeration from 2000 to 2020 were sourced from these statistical yearbooks. Data on energy consumption (2000–2020) cities within each urban agglomeration were sourced from national energy statistical yearbooks (China Energy Statistical Yearbook^36^, which were published by the National Bureau of Statistics of China (NBSC).

The data collection and processing analysis for various urban agglomerations in this study are based on municipal-level regional units. Municipal-level data can more accurately capture spatial heterogeneity and developmental disparities within an urban agglomeration. Taking the Pearl River Delta urban agglomeration as an example, although all its cities are located within Guangdong Province, significant differences exist in economic development levels, industrial structure, and population density among cities such as Guangzhou, Shenzhen, Zhuhai, and Foshan. Analysis at the municipal level effectively reveals such nuanced variations. In contrast, provincial statistical data often masks developmental disparities among cities within the same province. The use of municipal-level data helps avoid this “averaging effect” and more realistically reflects the developmental status of individual cities within an urban agglomeration^37^. For urban agglomerations spanning only a few provincial units, such as the Sichuan-Chongqing urban agglomeration—which involves only Sichuan Province and Chongqing Municipality—municipal-level analysis enables precise identification of each city’s specific contributions and functional roles in regional development, thereby circumventing the ambiguity inherent in provincial-level analysis. All statistical yearbooks provide municipal-level data compiled under unified statistical standards, ensuring comparability across cities and supporting reliable longitudinal and cross-sectional analyses.

To address missing statistical data in certain cities, this study employed two imputation methods: (1) for data missing continuously in time series, linear interpolation was applied; (2) for other missing data, an energy consumption-based estimation method was used, extrapolating values via the ratio of city-to-provincial energy consumption. Specifically, linear interpolation was applied to GDP data of Zigong City and Guang’an City from 2002 to 2004, and to population data of Xinyu City and Tianmen City from 2003 to 2005. The energy consumption estimation method was used to impute industrial output value data for Luzhou City and Yiyang City from 2000 to 2003. In terms of accuracy and sensitivity, the average relative error of linear interpolation was within 5%, while the error range of the energy consumption estimation method fell between 8% and 12%, with robustness confirmed via Monte Carlo simulation. All imputed total energy consumption values were cross-verified against provincial data to ensure consistency. These approaches ensured dataset completeness and reliability, establishing a solid data foundation for subsequent analysis.

### Evaluation of land-use carbon emissions and land-use carbon emission efficiency

#### Evaluation of carbon emissions from different land-use types

In this study, the land-use types of CulL, FL, GL, W, and UL are calculated using direct carbon emissions, as their area coefficients are relatively fixed^1,38^. The equation is as follows:

1 $${D_i}=\sum {{E_i} \cdot {\theta _i}}$$

where ${D_i}$refers to the land-use type i. ${E_i}$ denotes the area of land-use type *i*. ${\theta _i}$is the carbon emission fixed area coefficient for land-use type i, as shown in Table 3.

**Table 3:** Carbon emission fix area coefficients of land-use types^1,39^.

Land-use type	Coefficient(t/hm^2^)
CulL	0.422
FL	-0.644
GL	-0.022
W	-0.253
UL	-0.005

ConL’s carbon emissions are indirectly derived from energy consumption data covering multiple fuel types (e.g., coal, coke, fuel oil, gasoline, kerosene, diesel, liquefied petroleum gas, crude oil, and thermal energy) and electricity^40–42^. The equation is as follows:

2 $${E_b}=\sum\limits_{{i=1}}^{n} {{m_i} \cdot {n_i} \cdot {\varphi _i}}$$

where${E_b}$denotes the carbon emissions from ConL.${m_i}$represents the consumption of energy *i*.${n_i}$is the standard coal coefficient for the conversion of energy *i*. ${\varphi _i}$refers to the carbon emission coefficient of energy type *i*.

Energy-related carbon emission coefficients per standard coal unit were calculated using conversion factors from *China Energy Statistical Yearbook 2021*^31^ and emission parameters in the revised IPCC 2006 Guidelines^43^ (Table S2).

#### Evaluation of land-use carbon emission intensity

The land-use carbon emission (LUCE) per GDP is used to represent the regional LUCE intensity, as shown **in** Eq. 3.

3 $${I_i}=\frac{{{C_i}}}{{{G_i}}}$$

where ${I_i}$refers to the LUCE intensity of region *i*.${C_i}$denotes the LUCE of region *i*, and${G_i}$indicates the GDP of region *i*. The smaller value of ${I_i}$ indicates the lower intensity of LUCE. Higher values indicate greater economic impacts on LUCE, while lower values reflect achieved decarbonization through enhanced efficiency and green industry transitions.

### Integration of Logarithmic Mean Divisia Index model and Kaya Identity

This study employs Logarithmic Mean Divisia Index (LMDI) modeling (an enhanced approach integrating Kaya Identity principles^23^ with the context of China’s urban agglomerations) to analyze five key land-use carbon emissions determinants, including population size (PS), economic development (ED), energy structure (ES), energy consumption intensity (ECI), and industrial structure (IS). The LMDI method is applied to quantitatively assess the contribution of each factor to changes in the overall indicator, using the following equation:

4 $${\Delta _{LMDI}}=\sum\limits_{{i=1}}^{n} {{\alpha _i} \times (\log {{\rm X}_{it}}-\log {{\rm X}_{it-1}})}$$

where${{\rm X}_{it}}$represents the numerical values of each factor, and${\alpha _i}$represents the weighting coefficients. The sum of the weighting values for all factors equal to 1 and the weighting value of each factor is 1.

Combining the Kaya identity (carbon emissions = population × GDP × per capita carbon emissions) with the LMDI decomposition results, an extended model is formed:

5 $$C=POP \cdot \frac{{GDP}}{{POP}} \cdot \frac{C}{{ENERGY}} \cdot \frac{{ENERGY}}{{GDP}} \cdot {y_2}$$

where *C* refers to the LUCE of a region, *ENERGY* and *GDP* denote the regional total energy consumption and GDP, respectively, ${y_2}$indicates the GDP derived from the regional secondary industry, and *POP* represents the regional total population. By defining $p=POP$(population size), $g=\frac{{GDP}}{{POP}}$(economic development), $c=\frac{C}{{ENERGY}}$(energy structure), and $e=\frac{{ENERGY}}{{GDP}}$(energy consumption intensity), Eq. 5 can be transformed into a new form, namely Eq. 6.

6 $${C_j}=\sum\limits_{i} {{c_i} \cdot {g_i} \cdot {p_i} \cdot e{}_{i}} \cdot {y_{2i}}$$

where ${C_j}$represents the net LUCE of urban agglomeration *j*. The terms ${c_i}$,${g_i}$,${p_i}$,$e{}_{i}$, and ${y_{2i}}$ refer to the carbon emissions driven by the influencing factors of ES, ED, PS, ECI, and IS, respectively, in city *i*.

Furthermore, $C_{j}^{0}$ presents the carbon emissions of the base period in urban agglomeration *j*, $C_{j}^{T}$is the current carbon emissions in urban agglomeration *j*, the change in carbon emissions is defined by Eq. 7:

7 $$\Delta C_{j}^{T}=C_{j}^{T} - C_{j}^{0}=\sum\limits_{{i=1}}^{n} {{c_i}^{T} \cdot {g_i}^{T} \cdot {p_i}^{T} \cdot e{{{}_{i}}^T} \cdot y{{{}_{{2i}}}^T}} - \sum\limits_{{i=1}}^{n} {{c_i}^{0} \cdot {g_i}^{0} \cdot {p_i}^{0} \cdot e{{{}_{i}}^0}} \cdot y{{}_{{2i}}^0}$$

In the LMDI model, the contribution values of the influencing factors to LUCE are calculated by additive decomposing based on the Kaya Identity. The equations are as follows:

8 $$\Delta C_{j}^{{_{{{c_i}}}}}=\sum\limits_{{i=1}}^{n} {\frac{{C_{j}^{T} - C_{j}^{0}}}{{\ln C_{j}^{T} - \ln C_{j}^{0}}}} \times \ln \frac{{c_{i}^{T}}}{{c_{i}^{0}}}$$

9 $$\Delta C_{j}^{{_{{{g_i}}}}}=\sum\limits_{{i=1}}^{n} {\frac{{C_{j}^{T} - C_{j}^{0}}}{{\ln C_{j}^{T} - \ln C_{j}^{0}}}} \times \ln \frac{{g_{i}^{T}}}{{g_{i}^{0}}}$$

10 $$\Delta C_{j}^{{_{{{p_i}}}}}=\sum\limits_{{i=1}}^{n} {\frac{{C_{j}^{T} - C_{j}^{0}}}{{\ln C_{j}^{T} - \ln C_{j}^{0}}}} \times \ln \frac{{p_{i}^{T}}}{{p_{i}^{0}}}$$

11 $$\Delta C_{j}^{{{e_i}}}=\sum\limits_{{i=1}}^{n} {\frac{{C_{j}^{T} - C_{j}^{0}}}{{\ln C_{j}^{T} - \ln C_{j}^{0}}} \times \ln \frac{{e_{i}^{T}}}{{e_{i}^{0}}}}$$

12 $$\Delta C_{j}^{{{y_{2i}}}}=\sum\limits_{{i=1}}^{n} {\frac{{C_{j}^{T} - C_{j}^{0}}}{{\ln C_{j}^{T} - \ln C_{j}^{0}}} \times \ln \frac{{y_{{2i}}^{T}}}{{y_{{2i}}^{0}}}}$$

where $\frac{{C_{j}^{T} - C_{j}^{0}}}{{\ln C_{j}^{T} - \ln C_{j}^{0}}}$ is the logarithmic mean weight, and *n* is the number of cities in urban agglomeration *j*. The terms $\Delta C_{j}^{{_{{{c_i}}}}}$,$\Delta C_{j}^{{_{{{g_i}}}}}$,$\Delta C_{j}^{{_{{{p_i}}}}}$,$\Delta C_{j}^{{{e_i}}}$, and $\Delta C_{j}^{{{y_{2i}}}}$ denote the contribution values (i.e., the influencing effects on LUCE) of ES, ED, PS, ECI, and IS, respectively. Positive values signify factors driving emissions growth, while negative values indicate emissions-curbing effects.

### Markov chain method

The Markov chain model is used to predict events based on Markov chains, which describe each state transition depends only on the previous state and is independent of past status^44^. Specifically, the Markov chain model divides the studied dynamic systems into *n* possible states, identified as ${E_1},{E_2}, \cdots ,{E_{\mathrm{n}}}$. It then calculates the probability of transitions between these states, and constructs a state transition probability matrix^27^.

13 $$P={P_{ij}}=\left( {\begin{array}{*{20}{c}} {{P_{11}}}& \ldots &{{P_{1n}}} \\ \vdots & \ddots & \vdots \\ {{P_{n1}}}& \cdots &{{P_{nn}}} \end{array}} \right)$$

where ${P_{ij}}$indicates the transition probability from state ${E_i}$to state${E_j}$, and ${P_{ij}}$needs to meet the conditions: $0 \leqslant {P_{ij}} \leqslant 1$(*i*,* j* = *1*,*2…*,* n*), $\sum\limits_{{i=1}}^{n} {{P_{ij}}} =1$(*i* = *1*,*2…*,* n*). Given the initial state probability vector $E(0)$ and the state transition probability matrix *P*, the state probability distribution at any time *k* can be determined using Eq. 14:

14 $$E(k)=E(k - 1)P= \cdots =E(0){P^k}$$

where $E(k)$is the state probability vector at the time *k*, and $E(0)$ indicates the initial state probability vector.

The Markov chain model, as a data mining algorithm, relies on the probability transition matrix and the solution of equation systems^27^. Its computational process is straightforward and particularly suitable for scenarios with limited data. The algorithm is designed to enable efficient prediction and analysis when applied to small datasets. Consequently, the data scale in this study is appropriate for employing the Markov model. Furthermore, to validate the model’s prediction accuracy, cross-validation is adopted to evaluate its generalization capability. The data are partitioned into distinct subsets according to a three-year interval. The model is trained on the training set, and each validation set is sequentially used to assess performance. The average of all validation outcomes yields a prediction accuracy of 76%.

## Results

### Spatiotemporal evolution and characteristics of land-use carbon emissions

#### Land-use changes in urban agglomerations from 2000 to 2020

**Fig. 2:**
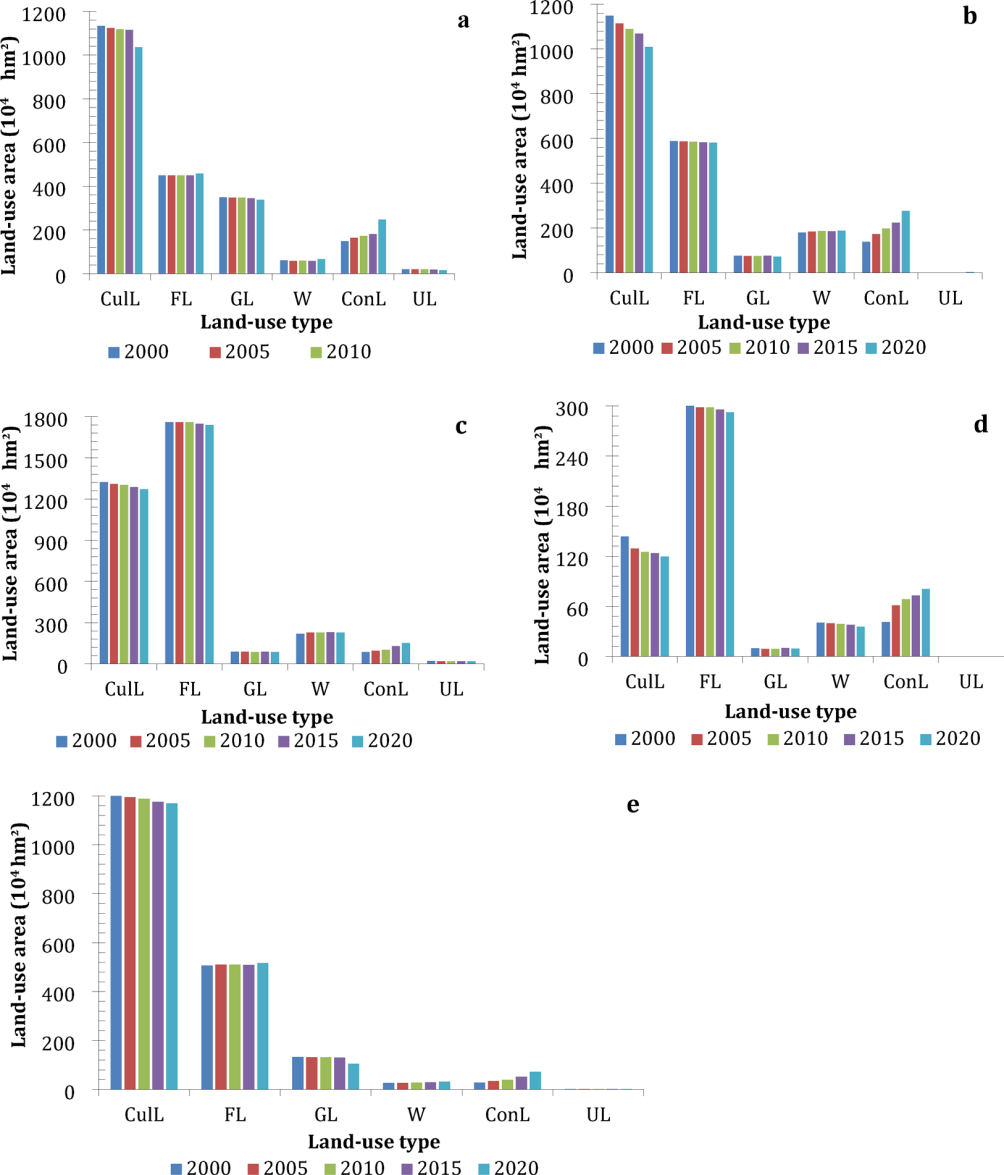
(**a**) Land-use changes in the BTH from 2000–2020; (**b**) Land-use changes in the YRD from 2000–2020; (**c**) Land-use changes in the MRYR from 2000–2020; (**d**) Land-use changes in the PRD from 2000–2020; (**e**) Land-use changes in the CC from 2000–2020.

Figure 2 illustrates the evolution of land area by different land-use types in the five major urban agglomerations from 2000 to 2020. The results show that cultivated land (CulL) and forest land (FL) are the dominant land-use types across these urban agglomerations. Specifically, FL accounts for the highest proportion in the PRD (Fig. 2d) and MRYR (Fig. 2c), followed by CulL. In contrast, the urban agglomerations of BTH (Fig. 2a), YRD (Fig. 2b), and CC (Fig. 2e) exhibit the opposite pattern, with CulL as the dominant land-use type.

From 2000 to 2020, construction land (ConL) experienced rapid growth in all five urban agglomerations, whereas CulL and grassland (GL) decreased. Additionally, the area of FL declined in the YRD, MRYR, and PRD urban agglomerations during this period. These land-use changes reflect the complex interplay among economic development, urbanization, policy interventions, and environmental factors. Rapid urbanization and economic growth have been the main drivers of construction land expansion, contributing to the reduction of agricultural and natural land areas. Such trends are especially evident in the YRD and PRD, which are among China’s most economically developed urban agglomerations.

As shown in Fig. 2a, from 2000 to 2020, the ConL in the BTH urban agglomeration increased by approximately 66%, which was the lowest growth rate among the five urban agglomerations. However, the absolute increase in ConL (about 99 × 10^4^ hm^2^) exceeded that of the PRD, MRYR, and CC. FL showed a slight increase, while CulL, GL, and unused land (UL) decreased significantly, by approximately 2 × 10^4^, 3 × 10^4^, and 11 × 10^4^ hm^2^, respectively. The relatively slow growth rate of ConL in the BTH region can be attributed to stricter land-use regulations and policies aimed at controlling urban sprawl in the capital area. Nevertheless, the substantial absolute increase reflects the region’s role as a national political and economic center, which drives demand for infrastructure and housing. The decline in CulL and GL is associated with urban expansion and land degradation, whereas the slight increase in FL may be attributed to afforestation initiatives designed to enhance air quality and ecological resilience.

Figure 2b illustrates that the YRD exhibited the most pronounced increase in ConL among the five urban agglomerations, expanding by nearly 100% (138 × 10^4^ hm^2^) between 2000 and 2020. Water bodies (W) and UL also increased slightly, whereas CulL, FL, and GL decreased significantly, by 138 × 10^4^, 6 × 10^4^, and 3 × 10^4^ hm^2^, respectively. Notably, the reduction in CulL was equivalent to the gain in ConL. The rapid expansion of ConL in the YRD reflects its position as the most economically dynamic region in China, a trend driven by industrialization, urbanization, and infrastructure development. The direct conversion of CulL into ConL underscores the trade-off between agricultural land and urban expansion. The slight rise in W may be attributed to water management initiatives, while the declines in FL and GL likely resulted from urban encroachment and the intensification of land use.

As shown in Fig. 2c, the MRYR exhibited a fluctuating decline in FL, GL, and UL from 2000 to 2020. In contrast, ConL increased substantially by 66 × 10⁴ hm² (a growth rate of 78%), whereas CulL showed a slight decrease. These land-use changes reflect the region’s transition from an agricultural-based economy to an industrial and urban economy. The rapid expansion of ConL was driven by regional development policies and infrastructure initiatives, such as the Yangtze River Economic Belt strategy. The fluctuating reductions in FL, GL, and UL indicate competing pressures from urbanization, agricultural modernization, and ecological conservation measures.

As illustrated in Fig. 2d, from 2000 to 2020, CulL, FL, and W in the PRD decreased by 24 × 10^4^, 10 × 10^4^, and 5 × 10^4^ hm^2^, respectively. During the same period, ConL expanded rapidly from 41 × 10^4^ hm^2^ to 80 × 10^4^ hm^2^, representing a growth rate of 96%. UL and GL also declined over this period. The rapid expansion of ConL in the PRD is attributed to its role as a global manufacturing hub and its integration with Hong Kong and Macao. The decrease in CulL and FL reflects the conversion of agricultural and forested land to urban and industrial uses. The reduction in W may be associated with land reclamation, while the declines in GL and UL are likely attributable to urban expansion and land degradation.

Figure 2e indicates that the CC recorded the highest growth rate of ConL among the five urban agglomerations, with a 1.62-fold increase (45 × 10^4^ hm^2^) from 2000 to 2020. FL and W increased by 10 × 10^4^ and 4.7 × 10^4^ hm^2^, respectively, while UL exhibited a marginal increase. In contrast, CulL and GL decreased significantly, by 34 × 10^4^ and 28 × 10^4^ hm^2^, respectively. The rapid expansion of ConL in the CC is attributed to its strategic role as a western economic hub and supportive government policies for regional development. The increases in FL and W reflect ongoing ecological restoration and water resource conservation measures. The substantial reductions in CulL and GL underscore the effects of urbanization and agricultural intensification, as well as potential challenges in reconciling economic growth with environmental sustainability.

#### Spatiotemporal evolution of land-use carbon emissions

The land-use carbon emissions and carbon sinks of these urban agglomerations from 2000 to 2020 were presented in Table S3. Notably, the net LUCE in all five urban agglomerations exhibited a turning point in 2010, after which it gradually increased from 2010 to 2020, as illustrated in Fig. S2.

A notable observation is the more than threefold in carbon emissions from ConL in the YRD, rising from 10914.85 × 10^4^ tons in 2000 to 42124.76 × 10^4^ tons in 2020 (Table S3). This marked growth reflects the rapid urbanization, industrialization, and economic development in the YRD, which have driven the expansion of ConL and the associated energy consumption.

In contrast, carbon emissions from CulL decreased across all five urban agglomerations from 2000 to 2020, consistent with the continuous decline in CulL area. This reduction is attributed to the conversion of agricultural land to urban and industrial uses, as well as improvements in agricultural practices that lower emissions.

The carbon sequestration effects of FL, GL, W, and UL remained relatively stable over the two decades. Among the five urban agglomerations, the MRYR exhibits the most pronounced carbon sequestration effect, with land-use carbon sinks reaching approximately 1179.21 × 10^4^ tons in 2020 (Table S3). This amount is nearly equivalent to the total carbon sinks of the other urban agglomerations, underscoring the MRYR’s significant role in carbon sequestration due to its extensive forest coverage.

Using the natural breaks (Jenks) method, a statistical classification technique based on numerical distribution patterns, the LUCE values of different cities within each urban agglomeration were categorized for the years 2000 and 2020. This method optimally partitions data into classes by minimizing intra-class variance and maximizing inter-class variance, thereby ensuring a meaningful representation of spatial patterns. Based on this classification, the spatiotemporal patterns of LUCE for each urban agglomeration are depicted in Fig. S3.

Figure S3a indicates that the number of cities with high LUCE in the BTH region decreased between 2000 and 2020. Among them, Tangshan maintained the highest LUCE values in both 2000 and 2020, which is consistent with its dominant coal and steel industries. In contrast, Shijiazhuang exhibited the most substantial decline in LUCE, despite its historical dependence on heavy industries such as steel and coal. The reduction in high-LUCE cities in the BTH region is likely due to regional policies aimed at industrial upgrading and environmental protection. The persistently high LUCE in Tangshan underscores the difficulty of transitioning energy-intensive industries, whereas the decrease in Shijiazhuang reflects the effective implementation of emission control measures and industrial restructuring.

As illustrated in Fig. S3b, in 2000, Shanghai and Ningbo were the main cities with high LUCE in the YRD. By 2020, the number of high-LUCE cities increased significantly, including marine port cities such as Ningbo and Zhousan, as well as cities along the Yangtze River, including Nanjing, Suzhou, and Wuxi. Consequently, the number of cities with low LUCE decreased. Notably, Shanghai’s LUCE declined substantially between 2010 and 2020, indicating its progress toward green and low-carbon development. The expansion of high-LUCE cities in the YRD reflects the region’s rapid economic growth, industrialization, and urbanization, especially in port and riverine cities. The reduction in LUCE in Shanghai is likely attributable to its advanced policies promoting renewable energy adoption, energy efficiency improvements, and sustainable urban planning, establishing a reference for other cities.

From 2000 to 2020, the number of cities with high LUCE in the MRYR increased significantly, predominantly along the mainstream of the Yangtze River (Fig. S3c), indicating a clear upward regional trend. This rise is primarily driven by industrial expansion and infrastructure development, especially within the Yangtze River Economic Belt. The trend is further exacerbated by less stringent emission controls and slower adoption of green technologies compared to coastal regions such as the YRD.

Figure S3d shows that from 2000 to 2020, the number of low-LUCE cities in the PRD increased. The city with the highest LUCE shifted from Guangzhou (the largest city in the PRD) in 2000 to Huizhou in 2020. Shenzhen, the second-largest city, experienced a decrease in LUCE from a secondary high level to a secondary low level. Moreover, the east-west disparity in LUCE became more pronounced, with higher levels observed in the east and lower levels in the west. The increase in low-LUCE cities in the PRD reflects achievements in industrial upgrading, technological innovation, and green urban development. Shenzhen’s reduction in LUCE can be attributed to its emphasis on high-tech industries and renewable energy adoption. The east-west disparity may result from uneven economic development and differences in the enforcement of environmental regulations.

As shown in Fig. S3e, from 2000 to 2020, Chongqing maintained a relatively high LUCE level, while Chengdu remained at a secondary high level. In contrast, the growth of LUCE in other cities slowed, leading to a notable rise in the number of low-LUCE cities, particularly in the northern part of the Chengdu-Chongqing (CC) region. Overall, the CC exhibited the most significant increase in low-LUCE cities among the five urban agglomerations. Chongqing’s high LUCE is associated with its industrial foundation and rapid urbanization, whereas Chengdu’s secondary high level reflects a balance between economic growth and environmental policies. The rise in low-LUCE cities, especially in the north, indicates the effectiveness of regional policies aimed at promoting sustainable development and reducing emissions.

#### Land-use carbon emission intensity and its spatial differentiation

Table S4 represents that from 2000 to 2020, both GDP and LUCE in each city exhibited a simultaneous growth trend. However, GDP growth significantly outpaced LUCE growth. As illustrated in Fig. S4, this disparity led to a substantial decrease in LUCE intensity across the urban agglomerations, indicating a trend toward green devolvement of economy. Furthermore, the differences in LUCE intensity among the five urban agglomerations have shown a narrowing trend, suggesting a convergence in carbon emission efficiency over time.

Although the value of LUCE intensity in the BTH decreased from 2000 to 2020, it remains the highest among the five urban agglomerations at 0.325 × 10^4^ t/100 million yuan. In 2020, cities with higher LUCE intensity were primarily distributed in the northern and southern parts of Hebei Province, with Handan and Tangshan recording the highest LUCE intensity at 1.03 × 10^4^ t/100 million yuan and 1.3 × 10^4^ t/100 million yuan, respectively. The high LUCE intensity in Hebei Province is largely due to its heavy reliance on energy-intensive industries such as steel and coal. In contrast, Beijing and Tianjin have optimized their industrial structures, transitioning to high-tech and high-end manufacturing sectors. Leveraging their population size and technological talent, these cities have significantly reduced their land-use carbon emission intensity, achieving the lowest LUCE intensity and ecological benefits in the BTH.

The YRD contributes nearly a quarter of China’s total economic output (GDP). However, its highly concentrated and active economic development has resulted in LUCE accounting for 40% of the total carbon emissions of the five urban agglomerations. Among the cities in the YRD, Zhoushan, a hub for high-end shipbuilding and marine engineering equipment manufacturing, recorded a GDP of 15.12 million yuan in 2020 but had the highest LUCE intensity in the region, with net LUCE reaching 3141 × 10^4^ tons. Similarly, Ma’anshan, a mining economy, had LUCE equivalent to Shanghai’s (1,627 × 10⁴ tons) but a GDP only 0.06 times that of Shanghai, reflecting its high energy dependency and high LUCE intensity. The high LUCE in Zhoushan and Ma’anshan underscores the challenges of energy-dependent economic growth. While the YRD’s economic dynamism drives national growth, its carbon intensity highlights the need for further industrial upgrading and energy efficiency improvements.

The MRYR encompasses three metropolitan regions, i.e., Wuhan, Changsha-Zhuzhou-Xiangtan, and Poyang Lake. In 2000, cities including Ezhou, Loudi, and Xinyu demonstrated substantially higher LUCE intensity (exceeding 0.5 × 10⁴ t/100 million yuan) than other regions, which was attributed to their established traditional industrial bases. Subsequent industrial restructuring has resulted in significant reductions in their LUCE intensity since 2000. Changsha exhibited the lowest LUCE intensity in the MRYR (0.023 × 10⁴ t/billion yuan), demonstrating superior energy utilization efficiency and minimal influence of economic expansion on LUCE. While industrial cities in the MRYR are progressively adopting more sustainable development approaches, their persistent dependence on conventional industries remains a constraint for further LUCE intensity reduction. Changsha’s exemplary low LUCE intensity provides a valuable reference for achieving equilibrium between economic development and carbon efficiency.

In the PRD, Huizhou exhibited the highest LUCE intensity in 2020 at 0.633 × 10⁴ t/100 million yuan. Conversely, Shenzhen, characterized by its advanced high-tech, financial, logistics, and cultural industries, recorded the lowest LUCE intensity (0.029 × 10⁴ t/100 million yuan). Dongguan, as a major manufacturing center, demonstrated substantial LUCE intensity reduction, achieving a decrease of 1.22 × 10⁴ t/100 million yuan between 2000 and 2020. Shenzhen’s minimal LUCE intensity reflects its sophisticated industrial structure and superior energy efficiency. Huizhou’s elevated LUCE intensity underscores the persistent challenges in energy-intensive manufacturing sectors, whereas Dongguan’s progress illustrates the effectiveness of industrial transformation initiatives.

In the CC, Chengdu, Deyang, and Zigong exhibited lower LUCE intensity. Neijiang, as a traditional industrial base, demonstrated significant improvement in LUCE intensity despite high energy consumption, achieving a reduction of 2.71 × 10⁴/100 million yuan from 2000 to 2020. Ya’an, characterized by its ecological advantages, maintained low energy consumption but limited GDP, resulting in the region’s highest LUCE intensity. The dual-core cities of Chongqing and Chengdu successfully transitioned to green, low-carbon energy consumption patterns, sustaining relatively low LUCE intensity levels. Neijiang’s progress reflects effective industrial restructuring, whereas Ya’an’s elevated LUCE intensity highlights the necessity for economic diversification to improve carbon efficiency.

### Influencing factors and spatiotemporal effects on land-use carbon emissions

#### Influencing factor decomposition of land-use carbon emissions based on LMDI model

**Fig. 3:**
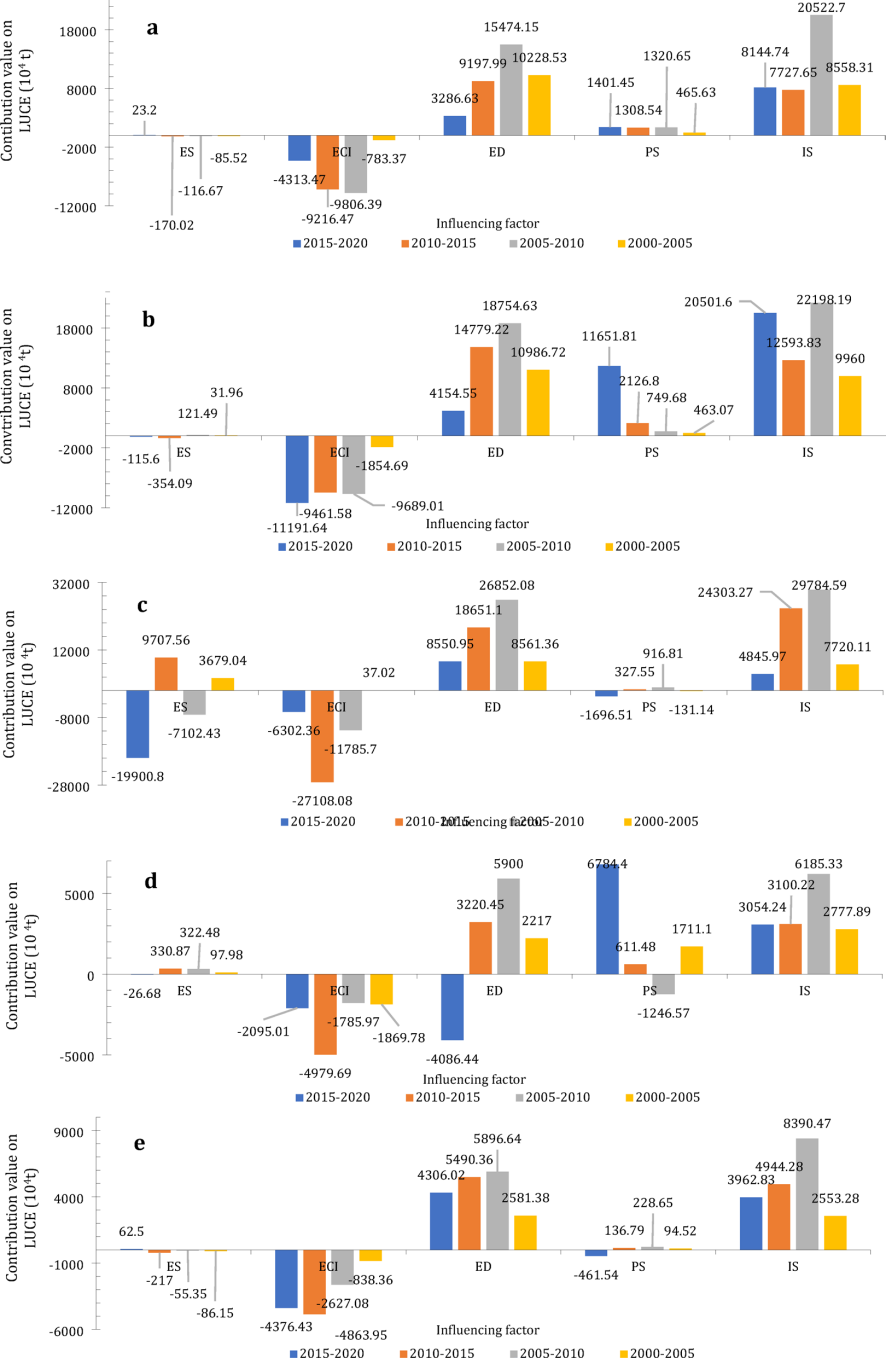
(**a**) Influencing factors’ contribution values on LUCE from 2000–2020 in the BTH; (**b**) Influencing factors’ contribution values on LUCE from 2000–2020 in the YRD; (**c**) Influencing factors’ contribution values on LUCE from 2000–2020 in the MRYR; (**d**) Influencing factors’ contribution values on LUCE from 2000–2020 in the PRD; (**e**) Influencing factors’ contribution values on LUCE from 2000–2020 in the CC.

Table 4; Fig. 3 present the contribution values (denoted as $\Delta C_{j}^{{_{{{c_i}}}}}$,$\Delta C_{j}^{{_{{{g_i}}}}}$,$\Delta C_{j}^{{_{{{p_i}}}}}$,$\Delta C_{j}^{{{e_i}}}$, and $\Delta C_{j}^{{{y_{2i}}}}$) of influencing factors—namely energy structure, economic development, population size, energy carbon intensity, and industrial structure—to LUCE from 2000 to 2020.

**Table 4:** Effects (contribution values) of influencing factors on LUCE from 2000–2020 (10^4^t).

UA	Period	$\Delta C_{j}^{{_{{{c_i}}}}}$	$\Delta C_{j}^{{{e_i}}}$	$\Delta C_{j}^{{_{{{g_i}}}}}$	$\Delta C_{j}^{{_{{{p_i}}}}}$	$\Delta C_{j}^{{{y_{2i}}}}$	$\Delta {C_{}}$
BTH	2015–2020	23.20	-4313.47	3286.63	1401.45	8144.74	397.81
	2010–2015	-170.02	-9216.47	9197.99	1308.54	7727.65	1120.04
	2005–2010	-116.67	-9806.39	15474.15	1320.65	20522.70	6871.74
	2000–2005	-85.52	-783.37	10228.53	465.63	8558.31	9825.27
YRD	2015–2020	-115.60	-11191.64	4154.55	11651.81	20501.60	4499.12
	2010–2015	-354.09	-9461.58	14779.22	2126.80	12593.83	7090.35
	2005–2010	121.49	-9689.01	18754.63	749.68	22198.19	9936.79
	2000–2005	31.96	-1854.69	10986.72	463.07	9960.00	9627.06
MRYR	2015–2020	-19900.80	-6302.36	8550.95	-1696.51	4845.97	-19348.72
	2010–2015	9707.56	-27108.08	18651.10	327.55	24303.27	1578.13
	2005–2010	-7102.43	-11785.70	26852.08	916.81	29784.59	8880.75
	2000–2005	3679.04	37.02	8561.36	-131.14	7720.11	12146.28
PRD	2015–2020	-26.68	-2095.01	-4086.44	6784.40	3054.24	576.28
	2010–2015	330.87	-4979.69	3220.45	611.48	3100.22	-816.89
	2005–2010	322.48	-1785.97	5900.00	-1246.57	6185.33	3189.93
	2000–2005	97.98	-1869.78	2217.00	1711.10	2777.89	2156.31
CC	2015–2020	62.50	-4376.43	4306.02	-461.54	3962.83	-469.45
	2010–2015	-217.00	-4863.95	5490.36	136.79	4944.28	546.20
	2005–2010	-55.35	-2627.08	5896.64	228.65	8390.47	3442.86
	2000–2005	-86.15	-838.36	2581.38	94.52	2553.28	1751.40

As shown in Fig. 3a, economic development (ED) and industrial structure (IS) were the primary drivers of LUCE growth in the BTH urban agglomeration from 2000 to 2020. The contribution of population size (PS) was relatively lower than that of ED and IS. This indicates the region’s dependence on industrial expansion and economic growth, which have resulted in increased carbon emissions. In contrast, energy consumption intensity (ECI) exerted a significant inhibitory effect on LUCE, as evidenced by its negative contribution value. However, the absolute contribution value of ECI gradually declined over time, indicating a diminishing inhibitory effect on LUCE. The weakening inhibitory role of ECI underscores the necessity of further enhancing energy efficiency to mitigate emission growth.

As shown in Fig. 3b, in the YRD, PS significantly drives LUCE, with its contribution value increasing steadily from 2000 to 2020, reaching 11651.81 × 10^4^ tons in 2020. In 2020, the average population density in the YRD was 657 people per square kilometer, making it a highly densely populated area in China. Between 2000 and 2020, cities such as Shanghai and Hangzhou experienced substantial population growth. Data from the Seventh National Census indicate that the combined population increase in these two cities reached nearly 14 million over this twenty-year period. Notably, Shanghai’s urban area recorded an average population density of 23,870.4 persons per square kilometer. Population growth emerged as the one of the dominant factors driving the increase in total LUCE. ED and IS also played major roles in promoting LUCE growth. Similar to the BTH, ECI exerted an inhibitory effect on LUCE, and its absolute contribution value increased over time, reflecting a strengthening inhibitory effect.

Figure 3c illustrates that in the MRYR, the influence of ES on LUCE exhibited temporal fluctuations, shifting from a driving effect (2000–2005) to an inhibitory effect (2005–2010), then reverting to a driving effect (2010–2015), and ultimately becoming inhibitory again (2015–2020). These fluctuations reflect changes in energy structure and policy interventions. ED and IS consistently served as the principal drivers of LUCE growth. In contrast, ECI exerted a notable inhibitory effect, although its influence diminished over time. The persistent driving roles of ED and IS highlight the region’s ongoing industrial and economic expansion, while the weakening inhibitory effect of ECI indicates a need for renewed emphasis on energy efficiency improvements.

As shown in Fig. 3d, in the PRD, IS and ES were the primary driving drivers for LUCE growth, with their contributions increasing from 2000 to 2020. IS constituted the dominant driving factor among all influences. Consistent with patterns observed in the BTH, YRDR, and MRYR, ECI exerted a suppressive effect on LUCE, especially between 2010 and 2015. ED exhibited a driving effect from 2000 to 2015 but shifted to an inhibitory effect during 2015–2020, indicating the diminishing role of high-energy-consuming industries in economic growth. These results indicate that the PRD’s industrial and energy structures have been major drivers of LUCE; however, the region’s transition toward a more service-oriented economy has started to alleviate emissions. PS also significantly contributed to LUCE growth during this period. According to the Seventh National Population Census, the PRD experienced a population growth rate exceeding 20% from 2000 to 2020, with Guangzhou and Shenzhen collectively increasing by 20 million residents. Furthermore, the urbanization rate in the PRD rose from approximately 55% in 2000 to over 85% in 2020, while population density in core cities like Guangzhou and Shenzhen increased by more than 40% during the same period. According to the China Energy Statistical Yearbook^2,36^, urban per-capita energy consumption grew by 15–20% in the PRD between 2000 and 2020. Consequently, the aggregate effect of population growth emerged as a dominant factor contributing to the increase in total LUCE.

According to Fig. 3e, in the CC, the driving and inhibitory effects of ES and PS on LUCE were relatively low compared to other urban agglomerations. The growth of LUCE was primarily influenced by IS and ED. Similar to other urban agglomerations, ECI exerted an inhibitory effect on LUCE, as indicated by its negative contribution value, which reflects its role in reducing emissions from 2000 to 2020. The reliance of the CC on IS and ED as driving factors corresponds to its industrial and economic development trajectory. The inhibitory effect of ECI underscores the region’s progress in enhancing energy efficiency, although further efforts are required to counteract the growth in emissions.

In conclusion, from 2000 to 2020, the influencing factors of ED and IS exerted significant driving effects on LUCE in the five urban agglomerations, whereas ECI demonstrated a distinct inhibitory effect. During this period, China underwent rapid urbanization and industrialization. The findings indicate that economic growth and energy consumption remain closely coupled. Achieving the “Dual Carbon” goals, decoupling economic development from energy consumption, and advancing green and low-carbon transition have become critical challenges for China’s new urbanization and industrialization. To address this challenge, it is necessary to gradually improve energy utilization efficiency, reduce energy consumption intensity, and achieve sustained reduction in land-use carbon emissions during urban agglomeration development. However, the analysis reveals that the impact of industrial structure on carbon reduction in urban agglomerations has been relatively weak, as the energy structure of these urban agglomerations did not undergo any fundamental changes from 2000 to 2020. To meet the “Dual Carbon” goals, a fundamental transformation of China’s energy structure should be prioritized. This transformation will determine whether clean energy can effectively replace fossil fuels and drive meaningful reductions in carbon emissions.

#### Cumulative decomposition contribution values of influencing factors

Based on the year 2000, the cumulative contribution values of various influencing factors—energy structure (ES), energy consumption intensity (ECI), economic development (ED), population size (PS), and industrial structure (IS)—on land-use carbon emissions (LUCE) in urban agglomerations was analyzed for the periods 2000–2005, 2000–2010, 2000–2015, and 2000–2020 (Fig. S5).

The cumulative contribution values of ECI in all five urban agglomerations were negative, indicating a consistent inhibitory effect on LUCE. ECI, defined as the ratio of energy consumption to GDP, reflects regional energy efficiency. Lower ECI values indicate higher energy efficiency, which contributes to restraining LUCE growth. Enhancing energy efficiency is essential for achieving economic growth without increasing total energy consumption, thereby improving the quality and efficiency of economic development.

From 2000 to 2020, ED exerted an increasingly significant driving effect LUCE growth in all urban agglomerations except the PRD. ED, measured as per capita GDP, reflects the level of regional economic development and prosperity. While rapid economic growth generates material wealth, it also leads to elevated LUCE. Therefore, future economic development must prioritize low-carbon strategies, balancing ecological conservation with economic growth to mitigate LUCE.

ES, defined as the ratio of total LUCE to energy consumption, had a relatively weaker influence on LUCE compared to other factors. A higher cumulative contribution value of ES indicates greater energy pollution intensity and increased LUCE. There remains substantial potential to reduce carbon emissions by optimizing the energy structure, particularly through the adoption of cleaner energy sources such as renewables, solar, and wind power.

PS drove LUCE growth in the PRD and YRD. These two regions are characterized by rapid population growth and high population density. Strategies should focus on electrification, construction of urban clean energy infrastructure, and promotion of low-carbon lifestyles.

IS, represented by added value of the secondary economic sector, exerted a significant cumulative increasing effect on LUCE. This sector is predominantly composed of traditional high-pollution industries, such as steel, mining, and machinery manufacturing. To achieve the “Dual Carbon” goals, it is essential to facilitate the transition of key industries toward cleaner and more efficient energy sources, while simultaneously optimizing industrial structures to mitigate emissions.

### Prediction of land-use carbon emissions based on Markov model

Land-use data from 2015 to 2020 for the five urban agglomerations were employed to analyze land-use transition probabilities, generate medium- and short-term predictions, and establish a stable transition probability matrix through cross-validation. A land-use stochastic matrix was constructed for each urban agglomeration on the ArcGIS platform. Based on this stochastic matrix, an initial state transition probability matrix was derived for each urban agglomeration (Table S6).

Taking 2020 as the initial state matrix and applying a five-year cycle, the Markov model was used to predict land-use changes in each urban agglomeration for 2025 (Table 5). The Markov model is suitable for projecting land-use changes to 2025 due to its short-term predictability, policy relevance, and data reliability. Its dependence on historical transition probabilities ensures higher accuracy over shorter periods, while the 2025 timeframe corresponds with *China’s 14th Five-Year Plan*, enabling direct applicability of the projections to current policy objectives.

**Table 5:** Prediction of land-use change and growth rate in urban agglomerations in 2025 (hm^2^).

UA		CulL	FL	GL	W	ConL	UL
BTH	2025	9,864,959	4,661,682	3,266,721	759,906	2,767,365	162,053
	2020	10,364,740	4,577,857	3,378,177	661,510	2,478,975	158,327
	Growth	-499,781	83,825	-111,456	98,396	288,390	3726
	Growth rate (%)	-4.82	1.83	-3.30	14.87	11.63	2.35
YRD	2025	9,557,029	5,800,295	679,611	1,884,648	3,014,580	48,036
	2020	10,092,422	5,818,920	725,181	1,885,424	2,765,385	34,031
	Growth	-535,393	-18,625	-45,570	-776	249,195	14,005
	Growth rate (%)	-5.30	-0.32	-6.28	-0.04	9.01	41.15
MRYR	2025	11,811,912	15,093,842	616,282	2,160,419	1,461,620	186,015
	2020	12,706,597	17,383,605	856,214	2,285,583	1,518,031	187,684
	Growth	-894,685	-2,289,763	-239,932	-125,164	-56,411	-1669
	Growth rate (%)	-7.04	-13.17	-28.02	-5.48	-3.72	-0.89
PRD	2025	1,164,320	2,991,160	100,710	329,664	870,256	500
	2020	1,196,989	2,921,205	95,255	355,067	8,062,917	667
	Growth	-32,669	69,955	5455	-25,403	63,965	-167
	Growth rate (%)	-2.73	2.39	5.73	-7.15	7.93	-25.04
CC	2025	11,503,572	5,796,168	1,304,917	307,581	865,202	27,105
	2020	11,692,674	5,164,523	1,044,743	316,380	722,215	18,480
	Growth	-189,102	631,645	260,174	-8799	142,987	8625
	Growth rate (%)	-1.62	12.23	24.90	-2.78	19.80	46.67

As shown in Table 5, ConL in all urban agglomerations except the MRYR is projected to increase from 2020 to 2025. Notably, ConL in the CC is expected to rise by 19.8%, while its CulL will decrease by 1.62%. Similarly, CulL in other urban agglomerations is also projected to decline during this period. Moreover, the increase in carbon source lands (mainly ConL) by 2025 is anticipated to surpass the growth of key carbon sink lands—GL, FL, and W. This indicates that ConL expansion is the principal driver of land-use changes across urban agglomerations. In contrast, carbon sink lands such as GL, FL, and W are not projected to increase substantially, highlighting the challenges in reconciling urban expansion with ecological conservation.

Based on the projected land-use cover changes, Table 6 and Fig. S6 present the evolution trends of land-use carbon emissions and carbon sinks in each urban agglomeration by 2025. The persistent expansion of construction land continues to pose a major challenge to low-carbon development, highlighting the necessity of industrial structure transformation, upgrading, and optimization.

**Table 6:** Prediction of land-use carbon emissions in urban agglomerations in 2025 (10^4^t).

UA	CulL	FL	GL	W	ConL	UL	Total
BTH	416.30	-300.21	-7.19	-19.23	31617.42	-0.08	31707.01
YRD	403.31	-373.54	-1.50	-47.68	44784.34	-0.02	44764.91
MRYR	498.46	-972.04	-1.36	-54.66	19641.17	-0.09	19111.48
PRD	49.13	-192.63	-0.22	-8.34	10192.31	0	10040.25
CC	506.55	-373.27	-2.87	-7.78	9987.84	-0.01	10110.46

To project regional LUCE intensity for 2025, we applied a linear regression model based on 2000–2020 GDP data for each urban agglomeration (Fig. S7). The 2025 GDP projections are presented in Table 7, which were used to calculate LUCE intensity in 2025. Table 7 shows that China’s “Dual Carbon” goals include an 18% reduction in LUCE intensity by 2025 relative to 2020 levels. However, the result reveals that none of the five urban agglomerations will achieve this target. Although the urban agglomerations of MRYR, BTH, and YRD demonstrate improvement, their reduction rates remain below the national target. The PRD and CC urban agglomerations exhibit particularly significant challenges, with the PRD’s LUCE intensity projected to increase by 3.26%.

**Table 7:** Land-use carbon emissions intensity in 2025 and 2020.

Year	Value	BTH	YRD	MRYR	PRD	CC
2025	GDP (predictive value) (100 million yuan)	107,134	237,059	110,883	105,451	78,132
	Net LUCE(predictive value) (10^4^t)	31,707	44,765	19,111	10,040	10,110
	LUCE intensity(10^4^t/100 million)	0.296	0.189	0.172	0.095	0.129
	Growth rate of LUCE intensity (%)	-8.92	-7.89	-6.52	3.26	0.00
2020	GDP(100 million yuan)	86,521	205,106	93,932	89,522	68,229
	Net LUCE(10^4^t)	28,129	42,126	17,181	8206	8813
	LUCE intensity(10^4^t/100 million)	0.325	0.205	0.184	0.092	0.129

### Carbon emission reduction policy pathways based on empirical findings

To address the identified challenges, targeted strategies with explicit implementation pathways are proposed for each urban agglomeration. For the BTH and MRYR, enhancing energy efficiency in heavy industries should prioritize the phase-out of outdated coal-dependent production capacities in sectors such as steel, while accelerating the deployment of waste heat recovery systems and green hydrogen-based direct reduced iron technologies. The transition to renewable energy should focus on developing distributed photovoltaic systems integrated with industrial parks and utilizing regional wind power bases to supply clean electricity for industrial processes. In the YRD and PRD, leveraging technological advances should involve implementing AI-driven energy management platforms and industrial internet systems to optimize energy use in electronics and automotive manufacturing. Scaling up solar and wind energy adoption requires expanding rooftop solar installations in manufacturing zones and offshore wind farms, supported by smart grid upgrades. For the CC, advancing clean energy projects should emphasize expanding hydropower capacity along the upper Yangtze River tributaries and deploying natural gas-fired peaking plants to enhance grid stability, while establishing green hydrogen production hubs in resource-rich areas like Sichuan to decarbonize the chemical and fertilizer industries.

Sustainable urbanization is essential, especially in the YRD and PRD, where rapid population growth is occurring. Sustainable urban planning must incorporate population migration trends into infrastructure development. In the PRD, this entails guiding labor-intensive industries and their workforces to relocate from core cities such as Guangzhou and Shenzhen to peripheral areas like Zhaoqing and Huizhou by enhancing regional transport connectivity and establishing specialized industrial parks supported by targeted housing policies for talent. Public transportation investments should prioritize cross-city metro systems and intercity high-speed rail to reduce vehicle emissions. Green infrastructure development should incorporate sponge city facilities and building-integrated photovoltaics. In the BTH and CC, balanced regional development requires transferring energy-intensive industries from Beijing and Chengdu to secondary cities such as Zhangjiakou and Mianyang, alongside incentives for high-value service sectors in core cities to alleviate population pressure. This strategy mitigates emissions from urban sprawl by fostering polycentric development patterns.

Industrial structure optimization is a critical priority for all urban agglomerations, necessitating sector-specific roadmaps. Financial incentives should be provided to promote clean and efficient energy technologies along with tax credits for carbon capture pilot projects in cement and chemical plants. For the MRYR and CC, transitioning traditional industries should focus on the food and light textile sectors in the CC by implementing circular economy models—such as utilizing agricultural waste for bioenergy production in Chongqing’s food processing clusters—and upgrading chemical industries in the MRYR through cogeneration and wastewater recycling.

Regional cooperation and policy integration are essential for realizing the “Dual Carbon” objectives. Urban agglomerations should establish technology-sharing platforms, including joint R&D centers for carbon capture technologies between the CC and BTH, and adopt standardized carbon accounting protocols consistent with international standards. Policy integration necessitates aligning regional industrial layouts with carbon reduction targets—for example, integrating the CC’s “Chengdu-Chongqing Hydrogen Corridor” initiative with national green hydrogen certification systems to facilitate cross-regional trading. Harmonizing regional development strategies with national goals also requires coordinated electricity market mechanisms that enable industries in the YRD, PRD, and MRYR to utilize renewable energy from solar and wind bases in western China through ultra-high-voltage transmission grids, thereby ensuring coherent implementation of decarbonization objectives.

The analysis of cumulative decomposition contributions reveals the interdependence among economic, demographic, and industrial drivers of LUCE. Addressing these challenges requires spatially explicit strategies integrating technological retrofitting, demographic management, and institutional innovation. Through the implementation of tailored pathways—such as industrial electrification in the BTH and population-guided infrastructure planning in the PRD—China’s urban agglomerations can achieve the “Dual Carbon” goals while sustaining economic resilience.

The predictions of land-use cover changes underscore the persistent challenges in managing land-use changes within China’s urban agglomerations. The continuous expansion of ConL and the reduction of CulL emphasize the necessity for sustainable land-use strategies that balance urban development with ecological conservation. Although the 18% reduction target of LUCE intensity by 2025 serves as a national average benchmark, the findings indicate persistent difficulties in lowering LUCE among urban agglomerations. These challenges arise from ongoing construction land expansion, dependence on energy-intensive industries, and inadequate advancement in transitioning toward low-carbon energy sources and industrial structures. Addressing these issues necessitates an integrated strategy that combines industrial transformation, optimization of energy structure, sustainable urban planning, and region-specific policies. By prioritizing these actions, urban agglomerations can make substantial progress toward fulfilling the “Dual Carbon” objectives and promoting sustainable development.

## Discussion

### A multi-regional framework for decarbonization policy enhancement

This study develops an integrated carbon accounting framework that integrates both direct energy emissions and construction land decomposition, advancing beyond traditional emissions-focused models^7,45^. Comparative analysis reveals marked regional disparities: the energy-intensive BTH urban agglomeration exhibits significantly higher carbon intensities than its southern counterparts, such as the YRD and PRD. Notably, the YRD and PRD exhibit enhanced carbon sink capacities through strategic industrial upgrades and energy structure optimization, indicating the need for region-specific emission reduction pathways.

Furthermore, this study integrates spatial-temporal pattern analysis with the identification of emission drivers, specifically addressing the regional variations emphasized in the decarbonization pathways of IPCC AR6^46^. The operational framework demonstrates distinct carbon neutrality approaches: the BTH requires technological upgrades, the YRD and MRYR demand spatial reconfiguration, while the CC and PRD need enhanced carbon sink management.

### Pathways for regional low-carbon transition

This study identifies four mutually reinforcing mechanisms for decarbonizing urban agglomerations. Compared with monocentric urban systems^47^, coordinated spatial planning emerges as the most effective approach, particularly through the following implementation strategies:

#### Spatial coupling of land use and industry

This study identifies industrial composition, particularly the expansion of secondary industries, as a more dominant driver of land conversion than demographic factors. The analysis indicates inadequate protection of carbon sinks, highlighting the need to enhance conservation of critical ecological spaces—such as forests, grasslands, and water bodies—that provide natural carbon sequestration. The findings underscore the disproportionate land-use impacts from energy-intensive secondary industries, calling for three key interventions: (1) establishing scientifically defined urban growth boundaries to balance development and ecological preservation; (2) implementing carbon sink management through restrictions on deforestation, optimized greening of marginal lands, and creation of ecological corridors; and (3) accelerating industrial transition by promoting clean energy adoption in heavy industries and facilitating technology-driven upgrades in traditional manufacturing sectors.

The proposed integrated approach addresses spatial planning and industrial restructuring simultaneously, providing a sustainable urbanization pathway that reconciles economic growth with environmental protection.

#### Synergistic spillover optimization

This study demonstrates that the five urban agglomerations should strengthen low-carbon synergies through three institutionalized spatial mechanisms, extending the concept of infrastructure synergy^48^. First, collaborative integration of resources and technology between central cities and surrounding areas enhances value chain efficiency while avoiding redundant investments. Second, multi-level governance necessitates standardized protocols for infrastructure, carbon accounting, and green finance to align economic and environmental objectives. Third, evolving leadership models toward collaborative platforms—such as cross-regional eco-industrial partnerships—support polycentric systems that balance decarbonization with growth imperatives. Subsequent development requires smart specialization strategies incorporating industry-specific spatial spillover analysis to optimize inter-city cooperation without undermining regional competitiveness.

#### Urban planning reforms for sustainable development

The spatial configuration of core economic zones, such as the PRD and YRD, significantly shapes land use and carbon emission patterns. This necessitates urban planning that emphasizes integrated low-carbon strategies spanning the energy, transportation, and industrial sectors. Meanwhile, policymakers should promote behavioral interventions to foster sustainable consumption patterns and long-term low-carbon lifestyles, thereby alleviating demand-side emission pressures.

#### Technology-governance co-evolution

Cross-regional analysis reveals distinct decarbonization pathways that necessitate adaptive governance-technology synergy. Technologically advanced urban agglomerations, such as the BTH, YRD and PRD, achieve success through cleantech ecosystems that integrate green innovation with industrial symbiosis, leading to substantial reductions in LUCE. In contrast, resource-intensive regions such as the CC and MRYR urban agglomerations achieve comparable decarbonization outcomes through land rehabilitation systems and ecological conservation. These findings extend conventional single-pathway LUCE theories^49^, demonstrating that effective decarbonization governance must balance two critical dimensions: facilitating cross-regional technology diffusion while maintaining local ecological carrying capacities.

## Conclusions

Studying LUCE is crucial for mitigating greenhouse gases and controlling global temperatures. In China, urban agglomerations serve as major economic growth engines yet face challenges in reconciling development with carbon reduction. This study examines LUCE in five major urban agglomerations across China, employing GIS, the LMDI model, and Markov chains to evaluate spatiotemporal patterns and influencing factors. The results reveal several key findings: (1) The PRD exhibited rapid LUCE growth driven by economic and population expansion, while growth slowed in the BTH, MRYR, YRD, and CC. (2) LUCEE improved across all urban agglomerations, with the PRD demonstrating the most advanced low-carbon practices. (3) Economic development and industrial structure were primary drivers of LUCE growth, while reductions in energy consumption intensity helped mitigate it. (4) Despite increased clean energy utilization, fossil fuels remained dominant, thereby limiting the contribution of energy structure to LUCE reduction. (5) By 2025, construction land expansion is projected to drive LUCE growth, with LUCE intensity declining in the PRD but improving in other regions.

This study advances LUCE research by analyzing spatiotemporal patterns, drivers, and regional variations, providing insights for policymakers to develop targeted carbon reduction and land-use planning strategies. Key limitations include: (1) reliance on five-year interval data from 2000 to 2020, which may obscure finer-scale LUCE dynamics; (2) LUCE calculations employing fixed coefficients for five land types and IPCC estimates for construction land, potentially improvable with additional energy data; and (3) insufficient analysis of resource coupling (water, soil, energy) and economic or policy impacts. Future research should address these gaps to enhance the comprehensiveness of LUCE understanding.

## Supplementary Information

Below is the link to the electronic supplementary material.

Supplementary Material 1

## Data Availability

The data used to support the findings of this study are available from the corresponding author upon request.
