# Supplementary material for: Influencing factors and spatiotemporal heterogeneity of land-use carbon emissions in China’s urban agglomerations
Source: Sci Rep. 2025 Dec 9;16:2083. doi: 10.1038/s41598-025-31817-1 (PMC12808698; doi:10.1038/s41598-025-31817-1)
Supplement: Supplementary file 1 — Supplementary Material 1 [file 41598_2025_31817_MOESM1_ESM.pdf]

Supplementary material

This supplementary material contains the figures (Fig. S1 to S7) and tables (Table S1 to S6) that have been cited in the manuscript text. Please refer to the cited numbers.

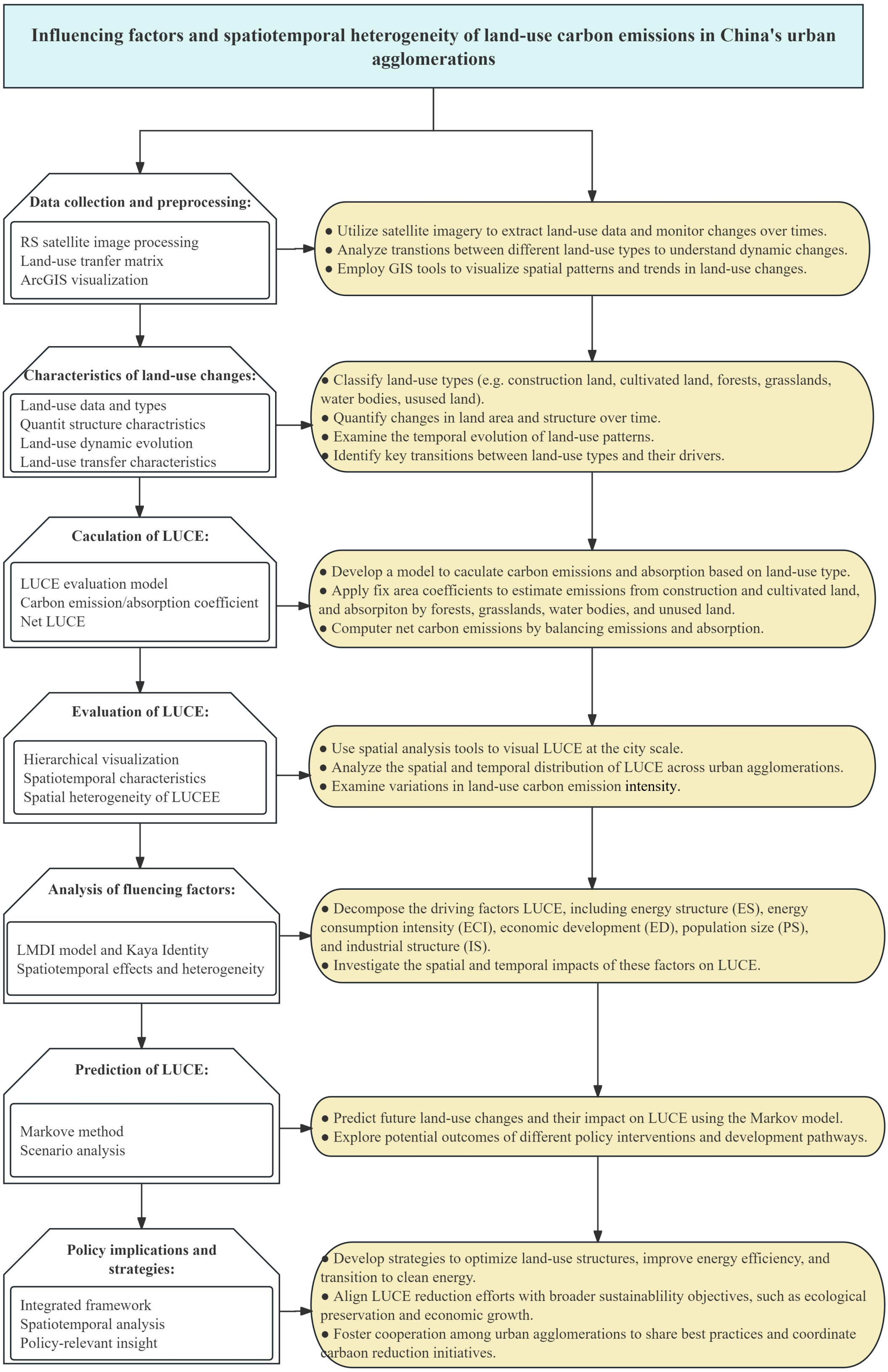

Fig. S1. Analysis framework

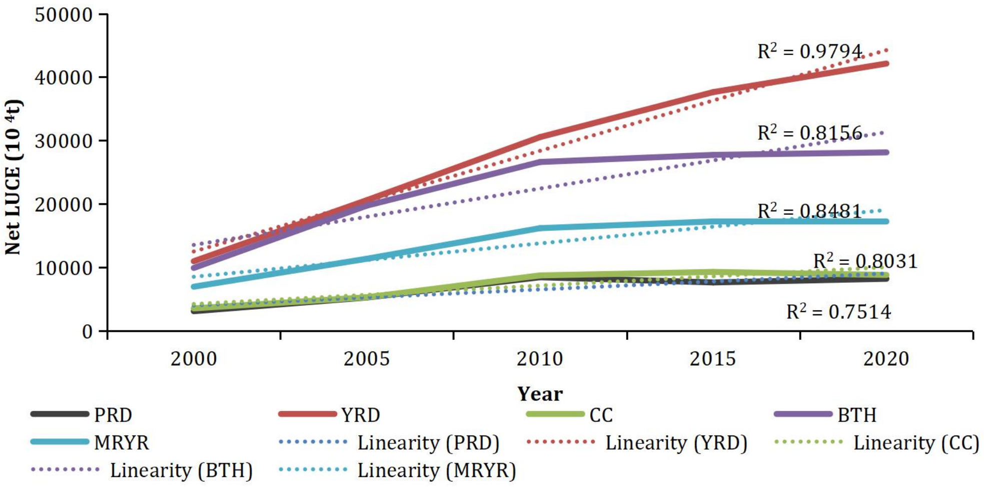

Fig. S2. Evolution of net LUCE in urban agglomerations from 2000 to 2020

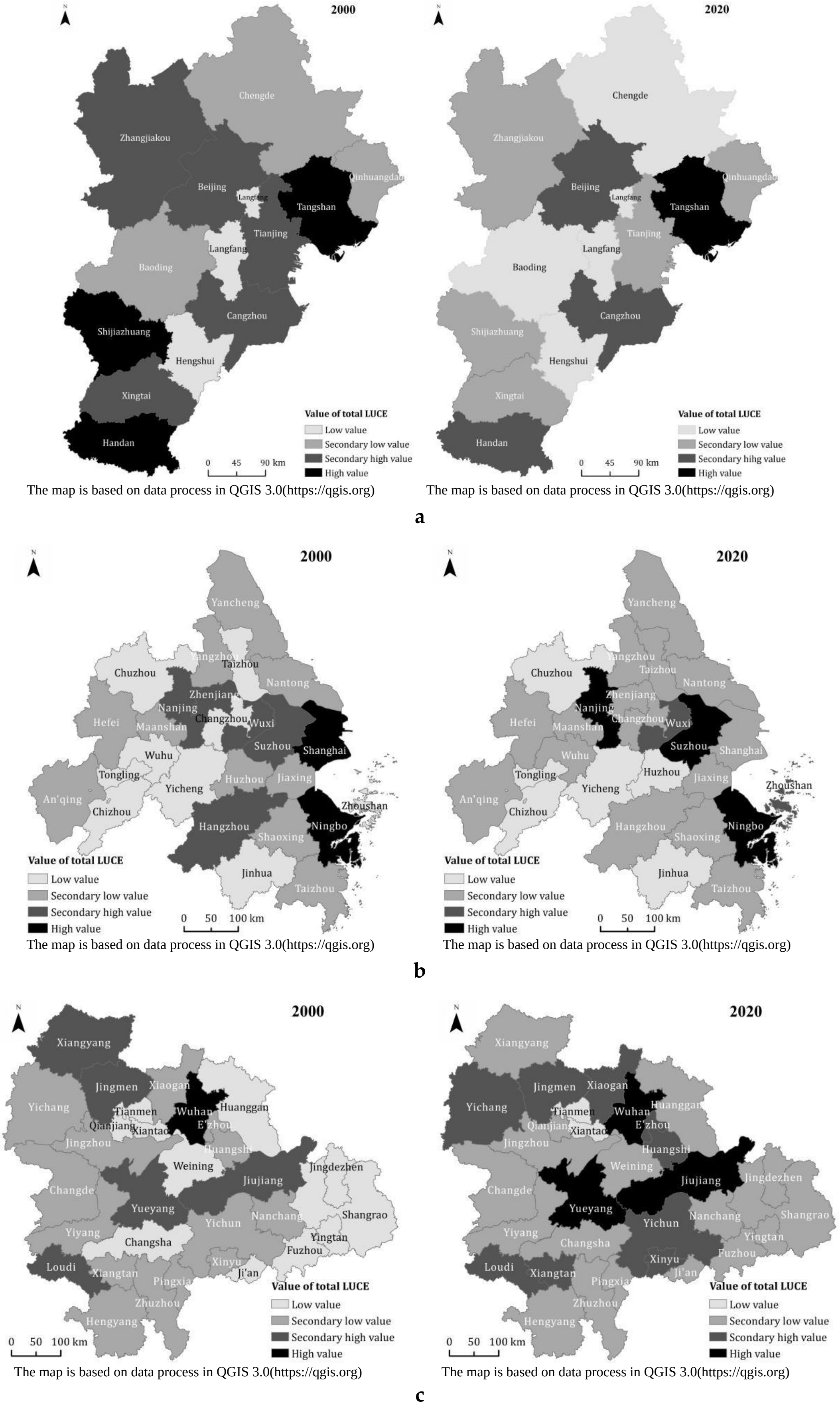
2000
Chengde
Zhangjiakou
Beijing
Langfang
Qinhuangdao
Tangshan
Tianjing
Langfang
Baoding
Shijiazhuang
Cangzhou
Hengshui
Xingtai
Handan
Value of total LUCE
Low value
Secondary low value
Secondary high value
High value
0 45 90 km
The map is based on data process in QGIS 3.0(https://qgis.org)
2020
Chengde
Zhangjiakou
Beijing
Langfang
Qinhuangdao
Tangshan
Tianjing
Langfang
Baoding
Shijiazhuang
Cangzhou
Hengshui
Xingtai
Handan
Value of total LUCE
Low value
Secondary low value
Secondary hihg value
High value
0 45 90 km
The map is based on data process in QGIS 3.0(https://qgis.org)
a
2000
Yancheng
Yangzhou
Taizhou
Chuzhou
Nantong
Zhenjiang
Nanjing
Changzhou
Hefei
Maanshan
Wuxi
Suzhou
Shanghai
Wuhu
Tongling
Yicheng
Huzhou
Jiaxing
An'qing
Zhoushan
Chizhou
Hangzhou
Ningbo
Shaoxing
Jinhua
Taizhou
Value of total LUCE
Low value
Secondary low value
Secondary high value
High value
0 50 100 km
The map is based on data process in QGIS 3.0(https://qgis.org)
2020
Yancheng
Yangzhou
Taizhou
Chuzhou
Nantong
Zhenjiang
Nanjing
Changzhou
Hefei
Maanshan
Wuxi
Suzhou
Shanghai
Wuhu
Tongling
Huzhou
Jiaxing
Zhoushan
Yicheng
An'qing
Chizhou
Hangzhou
Ningbo
Shaoxing
Jinhua
Taizhou
Value of total LUCE
Low value
Secondary low value
Secondary high value
High value
0 50 100 km
The map is based on data process in QGIS 3.0(https://qgis.org)
b
2000
Xiangyang
Jingmen
Xiaogan
Yichang
Tianmen
Wuhan
Huanggan
Qianjiang
Xiantao
E'zhou
Jingzhou
Huangshi
Weining
Jingdezhen
Jiujiang
Changde
Yueyang
Shangrao
Yichun
Nanchang
Yiyang
Changsha
Yingtan
Fuzhou
Loudi
Xinyu
Xiangtan
Ji'an
Pingxiang
Zhuzhou
Hengyang
Value of total LUCE
Low value
Secondary low value
Secondary high value
High value
0 50 100 km
The map is based on data process in QGIS 3.0(https://qgis.org)
2020
Xiangyang
Jingmen
Xiaogan
Yichang
Tianmen
Wuhan
Huanggan
Qianjiang
Xiantao
E'zhou
Jingzhou
Huangshi
Weining
Jiujiang
Jingdezhen
Changde
Yueyang
Yichun
Nanchang
Shangrao
Yiyang
Changsha
Yingtan
Fuzhou
Loudi
Xinyu
Xiangtan
Ji'an
Pingxiang
Zhuzhou
Hengyang
Value of total LUCE
Low value
Secondary low value
Scondary high value
High value
0 50 100 km
The map is based on data process in QGIS 3.0(https://qgis.org)
c

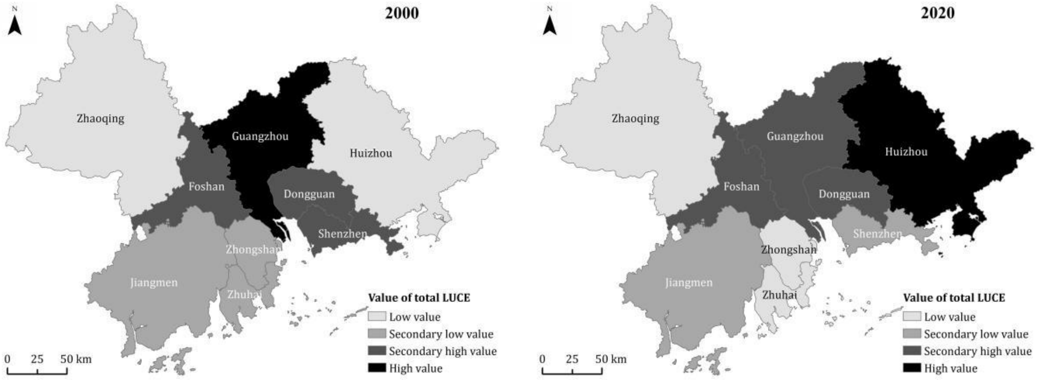

The map is based on data process in QGIS 3.0(https://qgis.org) The map is based on data process in QGIS 3.0(https://qgis.org)

d

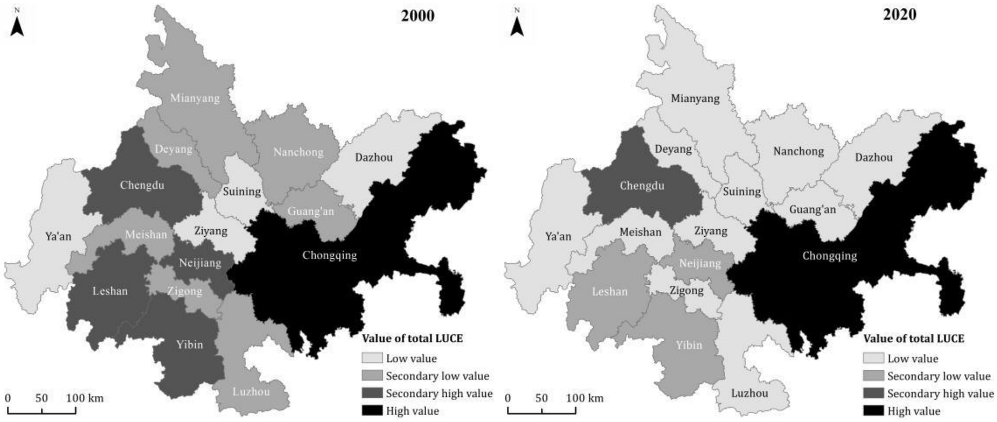

The map is based on data process in QGIS 3.0(https://qgis.org) The map is based on data process in QGIS 3.0(https://qgis.org)

e

Fig. S3. a) Spatiotemporal evolution characteristics of LUCE in the BTH (2000-2020); b) Spatiotemporal evolution characteristics of LUCE in the YRD (2000-2020); c) Spatiotemporal evolution characteristics of LUCE in the MRYR (2000-2020); d) Spatiotemporal evolution characteristics of LUCE in the PRD (2000-2020); e) Spatiotemporal evolution characteristics of LUCE in the CC (2000-2020)

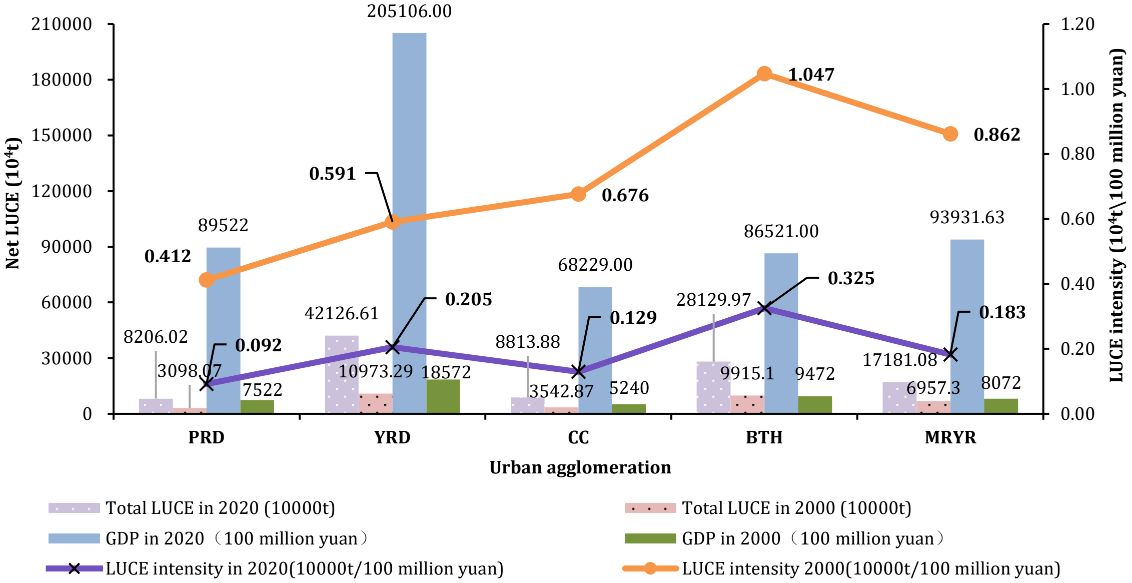

Fig. S4. Net LUCE and LUCE intensity across urban agglomerations in 2000 and 2020

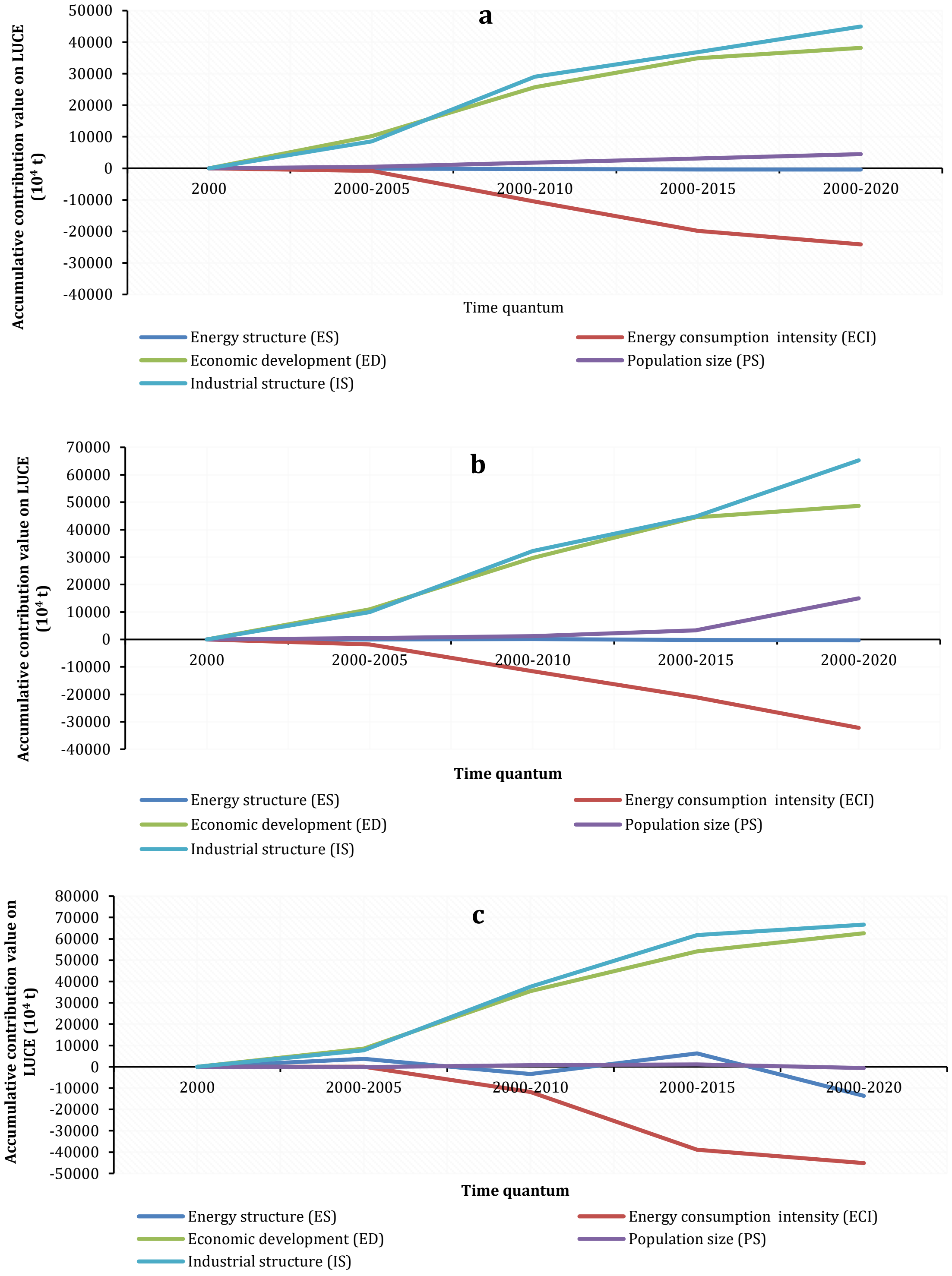
a
Accumulative contribution value on LUCE (10⁴ t)
50000
40000
30000
20000
10000
0
-10000
-20000
-30000
-40000
2000
2000-2005
2000-2010
2000-2015
2000-2020
Time quantum
Energy structure (ES)
Energy consumption intensity (ECI)
Economic development (ED)
Population size (PS)
Industrial structure (IS)
b
Accumulative contribution value on LUCE (10⁴ t)
70000
60000
50000
40000
30000
20000
10000
0
-10000
-20000
-30000
-40000
2000
2000-2005
2000-2010
2000-2015
2000-2020
Time quantum
Energy structure (ES)
Energy consumption intensity (ECI)
Economic development (ED)
Population size (PS)
Industrial structure (IS)
c
Accumulative contribution value on LUCE (10⁴ t)
80000
70000
60000
50000
40000
30000
20000
10000
0
-10000
-20000
-30000
-40000
-50000
2000
2000-2005
2000-2010
2000-2015
2000-2020
Time quantum
Energy structure (ES)
Energy consumption intensity (ECI)
Economic development (ED)
Population size (PS)
Industrial structure (IS)

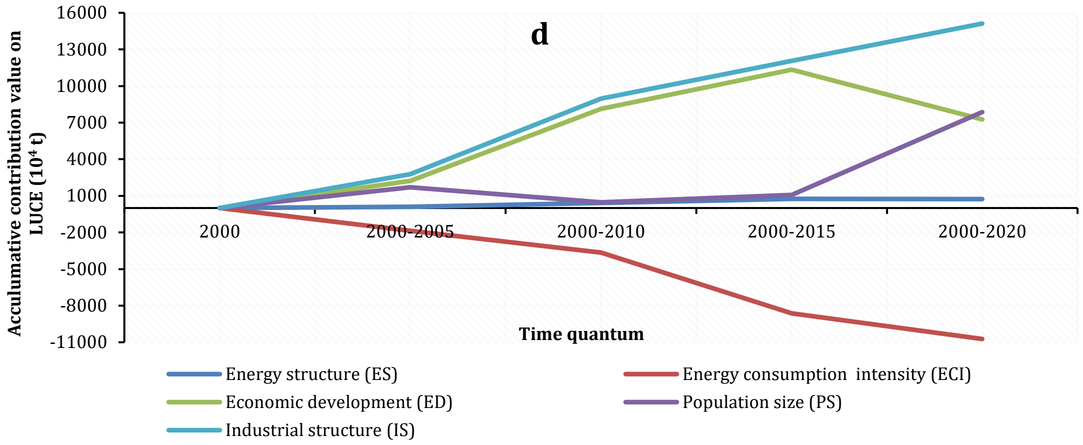

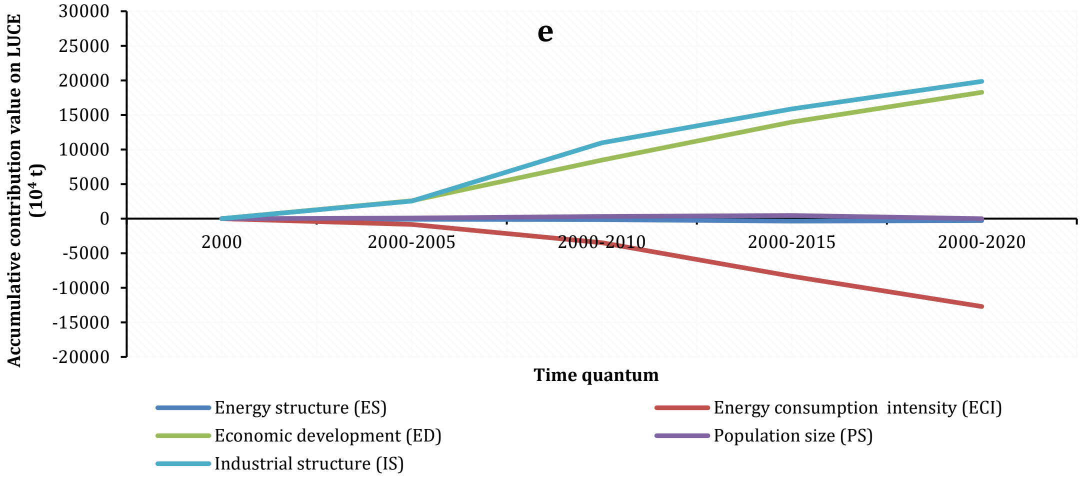

Fig. S5. a) Cumulative contribution values of different factors on LUCE in the BTH; b) Cumulative contribution values of different factors on LUCE in the YRD; c) Cumulative contribution values of different factors on LUCE in the MRYR; d) Cumulative contribution values of different factors on LUCE in the PRD; e) Cumulative contribution values of different factors on LUCE in the CC

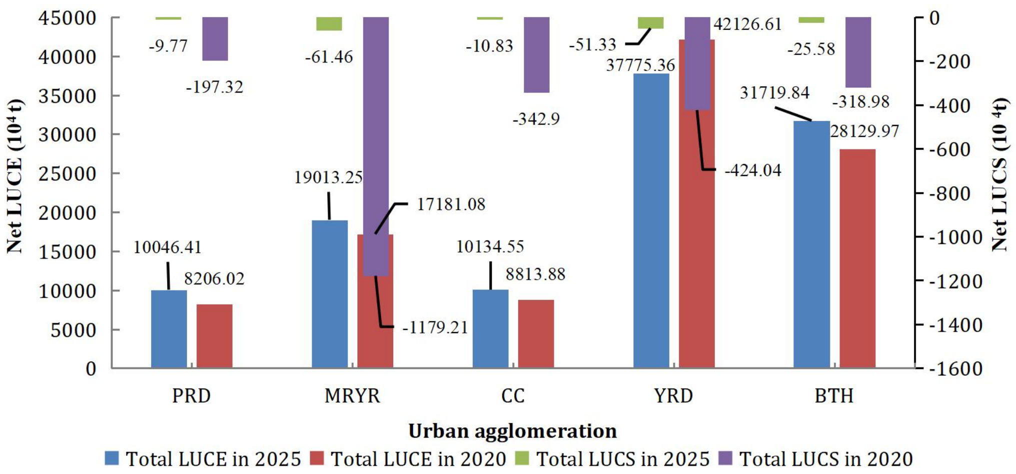

Fig. S6. Prediction of land-use carbon emissions and sequestration in urban agglomerations at 2020 and 2025

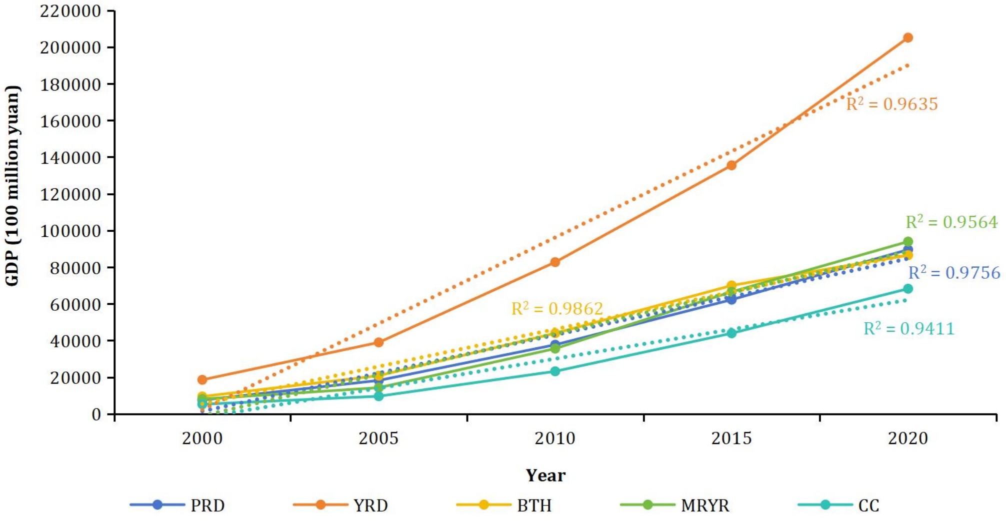

Fig. S7. Evolution of GDP of each urban agglomeration from 2000-2020

Table S1. Socio-economic statistics of the urban agglomerations in 2020

UA	Area (1×10^4 km^2)	Population (100 million)	GDP (100 million yuan)
BTH	22.2	1.1	9.78
YRD	22.5	1.65	27.6
MRYR	32.6	1.1	9.7
PRD	5.6	0.87	12.6
CC	18.5	1.03	7.6

Table S2. Conversion coefficients of energy standard coal and carbon emission coefficients

Energy	Conversion coefficient of standard coal	carbon emission coefficient
Coal	0.7143kg/kg	0.7559
Coke	0.9714kg/kg	0.8550
Fuel oil	1.4286kg/kg	0.6185
Gasoline	1.4714kg/kg	0.5538
Kerosene	1.4714kg/kg	0.5714
Diesel	1.4571kg/kg	0.5921
Liquefied petroleum gas	1.7143kg/kg	0.5042
Crude oil	1.4286kg/kg	0.5857
Thermal energy	0.03412kg/106J	0.7330
Electricity	0.1229kg/kWh	0.7476

Table S3. Land-use carbon emissions and sinks in urban agglomerations from 2000 to 2020（10^4 t）

UA	Year	CulL	FL	GL	W	UL	ConL	LUCS	Net LUCE
BTH	**2000**	478.40	-289.55	-7.69	-15.21	-0.10	9749.24	-312.45	9915.10
	**2005**	473.89	-289.46	-7.65	-14.77	-0.10	19578.46	-311.88	19740.37
	**2010**	471.85	-289.42	-7.63	-14.84	-0.09	26452.26	-311.89	26612.12
	**2015**	470.55	-288.98	-7.59	-14.60	-0.09	27572.88	-311.17	27732.16
	**2020**	437.39	-294.81	-7.43	-16.74	-0.08	28011.64	-318.98	28129.97
YRD	**2000**	484.38	-378.73	-1.66	-45.55	0.00	10914.85	-425.94	10973.29
	**2005**	469.80	-377.58	-1.65	-46.68	0.00	20556.47	-425.91	20600.35
	**2010**	459.73	-376.77	-1.64	-47.03	0.00	30502.86	-425.44	30537.14
	**2015**	451.00	-375.38	-1.67	-46.76	0.00	37600.31	-423.81	37627.49
	**2020**	425.90	-374.74	-1.60	-47.70	-0.02	42124.76	-424.04	42126.61
MRYR	**2000**	558.06	-1133.88	-1.96	-55.41	-0.10	7590.60	-1191.25	6957.30
	**2005**	552.58	-1132.80	-1.94	-57.81	-0.09	12004.07	-1192.55	11364.02
	**2010**	549.94	-1133.16	-1.89	-57.88	-0.09	16827.61	-1192.93	16184.52
	**2015**	542.74	-1125.56	-1.93	-58.27	-0.09	17884.62	-1185.76	17241.51
	**2020**	536.22	-1119.50	-1.88	-57.83	-0.09	17824.17	-1179.21	17181.08
PRD	**2000**	60.60	-194.63	-0.22	-10.25	0.00	3242.56	-205.10	3098.07
	**2005**	54.56	-192.10	-0.20	-10.02	0.00	5404.45	-202.32	5256.69
	**2010**	52.85	-191.95	-0.20	-9.81	0.00	8595.74	-201.96	8446.63
	**2015**	52.15	-190.31	-0.22	-9.54	0.00	7777.66	-200.07	7629.74
	**2020**	50.51	-188.13	-0.21	-8.98	0.00	8352.82	-197.32	8206.02
CC	**2000**	507.98	-326.04	-2.91	-6.83	-0.01	3370.68	-335.78	3542.87
	**2005**	504.03	-328.28	-2.90	-6.85	-0.01	5128.28	-338.03	5294.27
	**2010**	501.47	-328.55	-2.89	-7.07	-0.01	8574.17	-338.51	8737.13
	**2015**	496.22	-327.42	-2.88	-7.35	-0.01	9124.77	-337.65	9283.34
	**2020**	493.43	-332.60	-2.30	-8.00	-0.01	8663.36	-342.9	8813.88

Table S4. Land-use carbon emissions per unit GDP of urban agglomerations from 2000 to 2020

UA	Year	Net LUCE（10^4 t）	GDP / 100 million	LUCE-PUGDP/（10^4 t / 100 million）
	**2000**	9915.10	9472	1.047
	**2005**	19740.37	20847	0.947
**BTH**	**2010**	26612.12	43954	0.605
	**2015**	27732.16	69994	0.396
	**2020**	28129.97	86521	0.325
	**2000**	10973.29	18572	0.591
	**2005**	20600.35	38959	0.529
**YRD**	**2010**	30537.14	82716	0.369
	**2015**	37627.49	135513	0.278
	**2020**	42126.61	205106	0.205
	**2000**	6957.30	8072	0.862
	**2005**	11364.02	14293	0.795
**MRYR**	**2010**	16184.52	35595	0.455
	**2015**	17241.51	66551	0.259
	**2020**	17181.08	93932	0.183
	**2000**	3098.07	7522	0.412
	**2005**	5256.69	18244	0.288
**PRD**	**2010**	8446.63	37673	0.224
	**2015**	7629.74	62268	0.123
	**2020**	8206.02	89522	0.092
	**2000**	3542.87	5240	0.676
	**2005**	5294.27	9634	0.550
**CC**	**2010**	8737.13	23202	0.377
	**2015**	9283.34	43937	0.211
	**2020**	8813.88	68229	0.129

Table S5. Urban agglomeration land-use transfer matrix from 2015-2020（hm^2）

	BTH	2020					
Year	Type	CulL	FL	GL	W	ConL	UL
2015	CulL	8285007.50	465011.31	679907.57	213539.83	1363226.55	40435.28
	FL	361604.03	3383289.65	655936.50	26300.17	75522.64	3666.82
	GL	670478.62	676000.13	1873354.03	44039.45	119376.14	19581.26
	W	160904.09	35874.46	49216.44	205927.28	68063.75	42355.65
	ConL	720815.12	33186.25	49364.72	147129.10	824508.98	7331.58
	UL	76055.74	6969.47	30428.04	13211.96	12727.92	43656.74
	YRD	2020					
Year	Type	CulL	FL	GL	W	ConL	UL
2015	CulL	8011634.77	768984.84	110145.72	349736.63	1258482.11	5385.80
	FL	705348.61	4704219.36	190980.96	51793.62	115681.96	3520.18
	GL	119006.84	185056.74	347527.26	52478.70	22668.48	696.44
	W	324622.82	52128.32	31809.16	1266297.36	107429.88	19723.80
	ConL	758619.53	90585.30	17858.70	109626.85	1191876.50	2907.46
	UL	1029.04	1463.20	255.93	929.60	739.93	2174.45
	MRYR	2020					
Year	Type	CulL	FL	GL	W	ConL	UL
2015	CulL	8113308.55	2476969.74	117236.78	569671.31	651401.01	24210.02
	FL	2464482.10	12086286.52	270743.38	203580.41	220305.61	3000.34
	GL	113483.25	277186.58	205037.50	20095.79	12703.10	934.65
	W	583756.18	193510.04	16959.80	1233005.03	87492.42	66278.85
	ConL	513231.15	148456.71	9914.51	74093.73	397013.55	2274.78
	UL	22051.23	4248.80	1292.27	59639.36	1770.27	84464.82
	PRD	2020					
Year	Type	CulL	FL	GL	W	ConL	UL
2015	CulL	638956.13	293636.61	18747.47	102452.38	168927.41	140.85
	FL	284414.76	2514796.94	48293.57	54479.55	108123.28	0.37
	GL	18996.63	53588.27	24573.25	4148.92	6852.14	52.91
	W	116418.66	51565.37	3165.31	136299.24	64283.95	74.51
	ConL	127940.16	93644.71	7683.24	44719.65	467175.06	178.44
	UL	790.68	685.00	46.72	295.26	229.07	192.43
	CC	2020					
Year	Type	CulL	FL	GL	W	ConL	UL
2015	CulL	8502712.47	2295670.10	494701.00	188622.00	496323.77	6900.46
	FL	2383131.19	2855211.57	439257.59	48647.84	95597.37	10943.60
	GL	582954.97	514796.12	340340.51	13123.53	23289.61	3827.82
	W	173060.84	43161.52	10634.48	32119.30	27257.76	996.71

**ConL**	353792.45	78510.71	16133.92	24183.53	222036.64	417.62
**UL**	7919.90	8817.80	3849.19	884.85	696.73	4018.41

Table S6. Initial state transition probability matrix of urban agglomerations

UA	Type	CulL	FL	GL	W	ConL	UL
BTH	**CulL**	0.75	0.0421	0.0615	0.0193	0.1234	0.0037
	**FL**	0.0802	0.7508	0.1456	0.0058	0.0168	0.0008
	**GL**	0.197	0.1987	0.5505	0.0129	0.0351	0.0058
	**W**	0.2859	0.0637	0.0875	0.3659	0.1209	0.0753
	**ConL**	0.4043	0.0186	0.0277	0.0825	0.4625	0.0041
	**UL**	0.4155	0.0381	0.1662	0.0722	0.0695	0.2385
YRD	**CulL**	0.7627	0.0732	0.0105	0.0333	0.1198	0.0005
	**FL**	0.1222	0.8151	0.0331	0.009	0.02	0.0006
	**GL**	0.1636	0.2544	0.4777	0.0721	0.0312	0.001
	**W**	0.1801	0.0289	0.0176	0.7025	0.0596	0.0109
	**ConL**	0.3493	0.0417	0.0082	0.0505	0.5489	0.0013
	**UL**	0.1561	0.222	0.0388	0.141	0.1122	0.3299
MRYR	**CulL**	0.6788	0.2072	0.0098	0.0477	0.0545	0.002
	**FL**	0.1616	0.7926	0.0178	0.0134	0.0144	0.0002
	**GL**	0.1803	0.4404	0.3257	0.0319	0.0202	0.0015
	**W**	0.2677	0.0887	0.0078	0.5653	0.0401	0.0304
	**ConL**	0.4482	0.1297	0.0087	0.0647	0.3467	0.002
	**UL**	0.1271	0.0245	0.0074	0.3438	0.0102	0.4869
PRD	**CulL**	0.5225	0.2401	0.0153	0.0838	0.1381	0.0001
	**FL**	0.0945	0.8355	0.016	0.0181	0.0359	0
	**GL**	0.1755	0.4952	0.2271	0.0383	0.0633	0.0005
	**W**	0.3131	0.1387	0.0085	0.3666	0.1729	0.0002
	**ConL**	0.1726	0.1263	0.0104	0.0603	0.6302	0.0002
	**UL**	0.3531	0.3059	0.0209	0.1319	0.1023	0.0859
CC	**CulL**	0.8088	0.1234	0.0263	0.0124	0.0289	0.0002
	**FL**	0.2643	0.6622	0.0609	0.0045	0.0065	0.0016
	**GL**	0.249	0.2833	0.4537	0.0054	0.0061	0.0024
	**W**	0.4812	0.0798	0.0232	0.3194	0.092	0.0045
	**ConL**	0.3495	0.0528	0.0099	0.0346	0.553	0.0003
	**UL**	0.1425	0.2589	0.1497	0.0415	0.017	0.3905
